# Evaluation of Early Ketamine Effects on Belief-Updating Biases in Patients With Treatment-Resistant Depression

**DOI:** 10.1001/jamapsychiatry.2022.2996

**Published:** 2022-09-28

**Authors:** Hugo Bottemanne, Orphee Morlaas, Anne Claret, Tali Sharot, Philippe Fossati, Liane Schmidt

**Affiliations:** 1 Control-Interoception Attention Team, Paris Brain Institute, Sorbonne University, National Institute of Health and Medical Research, French National Centre for Scientific Research, Assistance Publique–Hôpitaux de Paris, Hôpital de la Pitié-Salpêtrière, DMU Neuroscience, Paris, France; 2 Department of Psychiatry, Pitié-Salpêtrière Hospital, DMU Neuroscience, Sorbonne University, Assistance Publique–Hôpitaux de Paris, Paris, France; 3 Department of Philosophy, Sorbonne University, SND Research Unit, UMR 8011, Paris, France; 4 Affective Brain Lab, Department of Experimental Psychology, University College London, London, United Kingdom; 5 Max Planck UCL Centre for Computational Psychiatry and Ageing Research, London, United Kingdom; 6 Department of Brain and Cognitive Sciences, Massachusetts Institute of Technology, Cambridge

## Abstract

**Importance:**

Clinical research has shown that persistent negative beliefs maintain depression and that subanesthetic ketamine infusions induce rapid antidepressant responses.

**Objective:**

To evaluate whether ketamine alters belief updating and how such cognitive effects are associated with the clinical effects of ketamine.

**Design, Setting, and Participants:**

This study used an observational case-control protocol with a mixed-effects design that nested 2 groups by 2 testing time points. Observers were not blinded. Patients with treatment-resistant depression (TRD) and healthy volunteer participants aged 34 to 68 years were included. Patients with TRD were diagnosed with major depressive disorder or bipolar depression, had a Montgomery-Åsberg Depression Rating Scale score greater than 20, a Maudsley Staging Method score greater than 7, and failed to respond to at least 2 prior antidepressant trials. Exclusion criteria were any other psychiatric, neurological, or neurosurgical comorbidities, substance use or addictive disorders, and recreational ketamine consumption. Data were collected from January to February 2019 and from May to December 2019, and data were analyzed from January 2020 to July 2021.

**Exposures:**

Patients with TRD were observed 24 hours before single ketamine infusion, 4 hours after the infusion, and 4 hours after the third infusion, which was 1 week after the first infusion. Healthy control participants were observed twice 1 week apart without ketamine exposure.

**Main Outcomes and Measures:**

Montgomery-Åsberg Depression Rating Scale score and belief updating after belief updating when patients received good news and bad news measured by a cognitive belief-updating task and mathematically formalized by a computational reinforcement learning model.

**Results:**

Of 56 included participants, 29 (52%) were male, and the mean (SEM) age was 52.3 (1.2) years. A total of 26 patients with TRD and 30 control participants were included. A significant group × testing time point × news valence interaction showed that patients with TRD updated their beliefs more after good than bad news following a single ketamine infusion (controlled for age and education: β = −0.91; 95% CI, −1.58 to −0.24; *t*_216_ = −2.67; *P* = .008) than controls. Computational modeling showed that this effect was associated with asymmetrical learning rates (LRs) after ketamine treatment (good news LRs after ketamine, 0.51 [SEM, 0.04]; bad news LRs after ketamine 0.36 [SEM, 0.03], *t*_25_ = 3.8; *P* < .001) and partially mediated early antidepressant responses (path a*b: β = −1.00 [SEM, 0.66]; *t*_26_ = −1.53; *z* = −1.98; *P* = .04).

**Conclusions and Relevance:**

These findings provide novel insights into the cognitive mechanisms of the action of ketamine in patients with TRD, with promising perspectives for augmented psychotherapy for individuals with mood disorders.

## Key Points

**Question:**

What are the effects of ketamine on belief updating in patients with treatment-resistant depression (TRD)?

**Findings:**

This case-control study in patients with TRD showed that belief updating became more optimistically biased as soon as 4 hours after a first ketamine infusion. This early cognitive effect of ketamine was formalized by stronger asymmetrical reinforcement learning and mediated at 1 week of treatment the clinical antidepressant effect.

**Meaning:**

These findings provide new perspectives for the understanding of the cognitive effects of fast-acting antidepressants that potentially can be leveraged to promote sustained clinical improvement and treatment responsiveness.

## Teaser

This case-control study evaluates whether ketamine alters belief updating and how such cognitive effects are associated with the clinical effects of ketamine.

## Introduction

Major depressive disorder (MDD) and bipolar depression are a crucial public health concern^1^ characterized by a range of negative beliefs, such as worthlessness, hopelessness, and pessimism.^2,3^ Cognitive models of depression propose that such maladaptive beliefs bias the perception and interpretation of life events and produce a negative view of oneself, the world, and the future.^3^ In addition to this cognitive triad, the excessively negative content of beliefs about the future^4^ involve a decreased sensitivity to disconfirming information.^5^ This phenomenon has also been referred to as pervasive pessimism^6^ and stands in stark contrast to the unrealistic optimism^7,8^ that is frequently observed in healthy individuals.

Cognitive studies have shown that beliefs about the likelihood of future events are updated after novel experiences.^9,10^ For healthy participants, belief updating is often asymmetric,^11,12,13^ with more belief updating following desirable than undesirable information. This effect has been termed the *good news/bad news bias* and is thought to underlie more general optimism biases^11^ that are crucial for maintaining mental and physical health.^13,14,15,16^ Importantly, patients with depression have been shown to lack an optimism bias in belief updating and to hold persistently negative expectations about the future, despite contradictory evidence.^17,18,19^ These characteristics are thought to play an important role in the maintenance of depressive symptoms and, potentially, in treatment resistance.^20,21,22,23^

Approximately one-third of patients with depression do not respond to conventional antidepressant treatments and thereby experience treatment-resistant depression (TRD).^24^ Over the last decade, ketamine, an ionotropic glutamatergic *N*-methyl-d-aspartate receptor antagonist, has become an exciting antidepressant therapy for TRD.^25^ Several meta-analyses of placebo-controlled randomized clinical trials have shown that ketamine has a rapid antidepressant effect that peaks within 24 hours.^26,27^ Despite these promising results, little is known about the cognitive effects of ketamine in TRD and their link to clinical improvement.

Interestingly, pharmacological studies on healthy participants have shown that ketamine disturbs the belief-updating process by changing the way participants update their beliefs when faced with new information.^28,29,30^ Moreover, another study on MDD has shown that a single ketamine infusion can produce a sustained improvement in depressive beliefs.^30^ However, it is unknown how ketamine affects the mechanisms of belief updating in patients with TRD, which are potentially key to understanding its antidepressant effects.

Here, we asked (1) whether and through what computational mechanisms ketamine restores optimism biases in belief updating and (2) how such potential cognitive effects link to antidepressant effects. We tested these hypotheses by following a measurement-based care approach, which consisted of objectifying the clinical assessments and outcomes of patients undergoing ketamine treatment.^31^ We combined this approach with an observational assessment of clinical improvement and belief-updating behavior and then mathematically formalized observed effects by using a computational model inspired by reinforcement learning (RL) principles^32^ and mediation analysis.

## Methods

### Participants

The study was conducted in accordance with the Declaration of Helsinki and was approved by the local authorities at the Pitié-Salpêtrière Hospital. Participants gave oral informed consent prior to participating in the experiment. This study followed the Strengthening the Reporting of Observational Studies in Epidemiology (STROBE) reporting guideline.

In total, 26 patients with TRD and 30 healthy control participants were enrolled in the study (eTable 1 in the Supplement). The sample size was determined to be consistent with those of prior studies of belief updating in patients with depression and by the reality of patient recruitment in the clinical setting.^17,18^

Healthy participants were recruited at the Pitié-Salpêtrière Hospital campus. Patients were recruited at the Psychiatry Department of the Pitié-Salpêtrière Hospital and were clinically evaluated by a psychiatrist specialized in TRD during a clinical interview to assess the inclusion and exclusion criteria. Patient inclusion criteria included age between 18 and 70 years, MDD or bipolar depression according to the *DSM*-*5* criteria, a Montgomery-Åsberg Depression Rating Scale (MADRS) score greater than 20, TRD (defined by the failure to respond to at least 2 antidepressant treatments), and moderate to high treatment resistance, as defined by the Maudsley Staging Method (MSM) score greater than 7 (eTable 2 in the Supplement).^33,34^

Exclusion criteria for all participants were psychiatric disorders other than TRD, neurological and neurosurgical comorbidities, substance use or addictive disorders in the last 12 months, and previous recreational ketamine consumption. All participants were able to understand and perform the belief-updating task.

### Study Design

**Figure 1.**
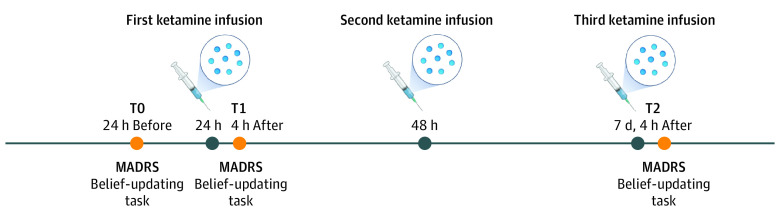
Study Design Patients were administered a total of 3 doses of ketamine (0.5 mg/kg) by intravenous infusion over the course of a week. Depression (measured by Montgomery-Åsberg Depression Rating Scale [MADRS]) and belief updating (measured by the belief-updating task) were assessed at 3 times: 24 hours before (T0) and 4 hours after (T1) the first infusion and 4 hours after (T2) the third ketamine infusion, which occurred 1 week after the first infusion. At each time point (T0, T1, and T2), testing lasted for a total duration of 1 hour.

The study was an observational case-control study with a mixed-effects design that nested 2 factors: group and testing time points (Figure 1).

### Ketamine Treatment Administration

Ketamine treatment was administered in the standard clinical care setting following a previously reported procedure^35^ (eMethods 1 in the Supplement). It was open labeled, and the clinical staff, who administered the drug, was not blind to the treatment arm. Patients were also not randomly assigned to the treatment.

### Principle and Secondary Outcome Measures

The principal outcome measures were belief updating and clinical improvement. Belief updating was measured by the difference between an initial belief estimate and a second belief estimate given after being provided with information about the actual base rates of experiencing a given adverse life event (eMethods 2 in the Supplement). Clinical improvement in depressive symptoms was assessed using the MADRS.^36^ Secondary outcome measures involved learning rates (LRs) obtained from a computational RL-like model, remission (ie, greater than 50% improvement and a MADRS score of 10 or less), treatment responsiveness (ie, greater than 50% improvement and a MADRS score greater than 10), and prognostic outcome expectancies (eMethods 2 and 3 in the Supplement).

### Belief-Updating Task

**Figure 2.**
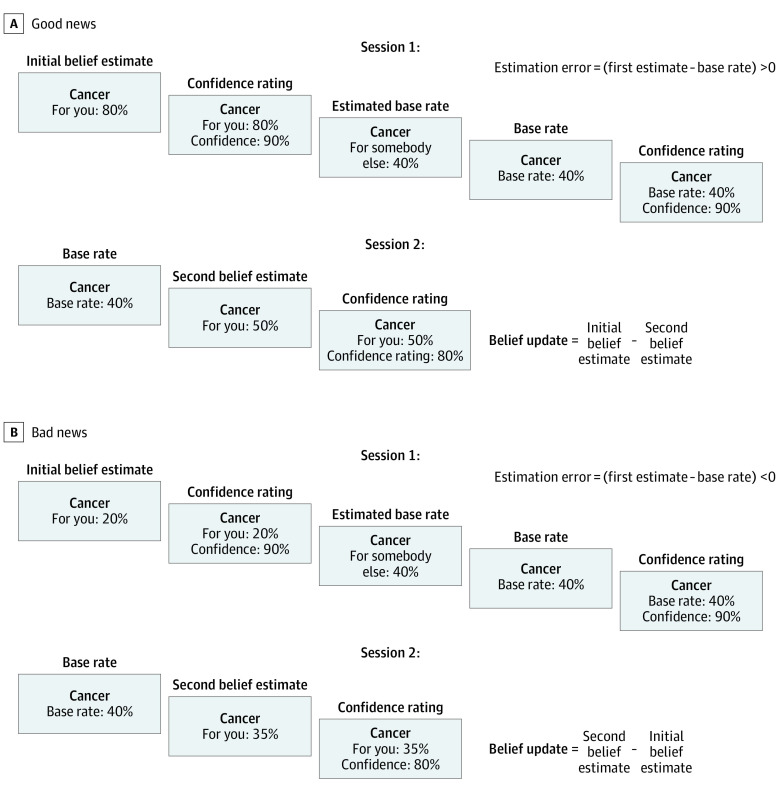
Behavioral Task Screenshots show successive events for 2 example trials, one for good news (A) and one for bad news (B). The task was self-paced, and individual response times were recorded. The task design comprised 2 sessions. During the first session, participants were presented with an adverse life event for each trial and were asked to estimate their own as well as other people’s likelihood of experiencing that event in the future (initial belief estimate and estimated base rate, respectively). They also rated their confidence in each estimate. At the end of each trial, the average likelihood of that event occurring in the general population (base rate) was displayed, and participants rated their confidence in that base rate. During the second session, each trial started with the presentation of the actual base rate associated with an event, and participants reestimated their likelihood of experiencing this event in the future (second belief estimate) as well as how confident they were in their second estimate.

All participants performed a belief-updating task adapted from decision neuroscience^17,18,32,37,38^ (Figure 2). The task involved 2 sessions of 40 trials. In session 1, participants were asked on a trial-by-trial basis to estimate their lifetime likelihood of experiencing varying adverse life events (ie, the initial estimate) and the likelihood of someone else with a similar socioeconomic background experiencing a given event (ie, the estimated base rate). At the end of each trial, the participants got to know the actual base rate of events in the general population. If the actual base rate was smaller than the participant’s initial estimate, the trial was categorized as a good news trial. If the actual base rate was greater than the participant’s initial estimate, the trial was categorized as a bad news trial. The participant’s attention was not explicitly drawn to this good news/bad news categorization of the trials. Importantly, the same 40 adverse life events were presented again in session 2, and again on a trial-by-trial basis participants reestimated their lifetime risk, now taking into account the base rate. See eMethods 4 and 9 in the Supplement for details on task variables and for detailed task instructions.

### Statistical Analysis

All statistical tests (eg, linear mixed-effects models, χ^2^, Wilcoxon signed rank test, *t* tests, nonparametric permutation, and bootstrap tests) were conducted using the Statistics and Machine Learning Toolbox (MATLAB version 2015a; MathWorks), and the Mediation Toolbox.^39,40^ Computational modeling was implemented using the using the VBA toolbox.^41,42^ Two-sided *P* values were statistically significant at less than .05. 

#### Statistical Analysis of Global Clinical Improvement

Two-tailed signed Wilcoxon rank tests were used to assess statistically significant effects of treatment on the MADRS scores between baseline (T0), 4 hours after the first ketamine infusion (T1), and 1 week after the first infusion (T2).

#### Statistical Analysis of Belief Updating

A linear mixed-effects model fitted absolute belief updating (|UPD|) using the following equation: |UPD| ∼ 1 + Group + Time + valence EE + |EE| + Age + Education + (Group × Time × valence EE) + (1 | Subject) + (1 + valence EE | Subject) + (1 + |EE| | Subject). The model included fixed slopes for the effects of estimation error valence (valence EE; coded 1 for good news, −1 for bad news), group (coded 1 for healthy controls, −1 for patients with TRD), testing time point (coded −1 for T0 at baseline, 1 for T1 4 hours after the first infusion), absolute EE magnitude (|EE|), age, years of higher education, and the 3-fold interaction of interest, group × valence EE × time. The model also included random intercepts grouped by participant number and random slopes for valence and absolute EE (|EE|) by participant. See eMethods 5 and eTable 5 in the Supplement for more details on belief updating task variables and eTable 12 in the Supplement for results of an analoguous model fitted to belief updating bias.

The *R*^2^ scores for this model were 0.7179 ordinary and 0.708 adjusted. Trials with zero EEs were excluded, because they did not leave any room for updating. Moreover, trials in which the estimates of the participants increased despite good news and decreased despite bad news were removed from the analyses (eTable 4 in the Supplement). Analyses did lead to similar results when including these paradoxical trials (eMethods 6 and eTable 13 in the Supplement).

#### Computational Modeling of Belief Updating

To gain insights into the cognitive mechanisms underlying the effects of ketamine on belief updating, we used a computational model that has previously been reported to best explain optimistically biased belief updating.^32,38^ The model relied on a generic RL algorithm that assumed belief updating is proportional to the size of the EE, which is weighted by the LR (eMethods 7 in the Supplement). Eight possible variations of the RL model were compared in a 2 × 2 × 2 design to test how much keeping 2 components of the LR (ie, the alpha and asymmetry component) free or at zero, and whether further weighting by the personal relevance of events played a role. Two criteria were considered for model selection: the estimated model frequency in each cohort and the exceedance probability, which corresponds to the probability of the model to be above chance in each cohort.^32^ For more detail on the RL model, see eMethods 7 and eTables 17 and 18 in the Supplement.

#### Mediation Analysis

The mediation model tested the null hypothesis that the effect of ketamine on the belief-updating bias and the effect of the belief-updating bias on MADRS score were uncorrelated (eMethods 2 in the Supplement).^43^ A bootstrap test was used to infer significant mediation effects. This involved calculating a distribution of individual path coefficients of the mediation model based on 10 000 random samples, with the replacement of observed coefficients. Then, bilateral *P* values were calculated based on the confidence intervals of the distribution of bootstrapped coefficients. For more detail on the mediation analysis, see eMethods 8 in the Supplement.

## Results

### Clinical Response

Of 56 included participants, 29 (52%) were male, and the mean (SEM) age was 52.3 (1.2) years. A total of 26 patients with TRD and 30 controls were included. Ketamine treatment induced a rapid and significant decrease in the MADRS scores 4 hours after a first ketamine infusion (*z* = 3.33; *P* = .001), which remained significantly lower than baseline scores one week later (*z* = 4.1; *P* < .001) (eTable 3 in the Supplement). One patient with TRD went into remission, and 5 showed a treatment responsiveness at 1 week after treatment (eMethods 2 and 3 and eFigure 1 in the Supplement).

### Outcomes of Ketamine on Optimism Biases in Belief Updating

**Figure 3.**
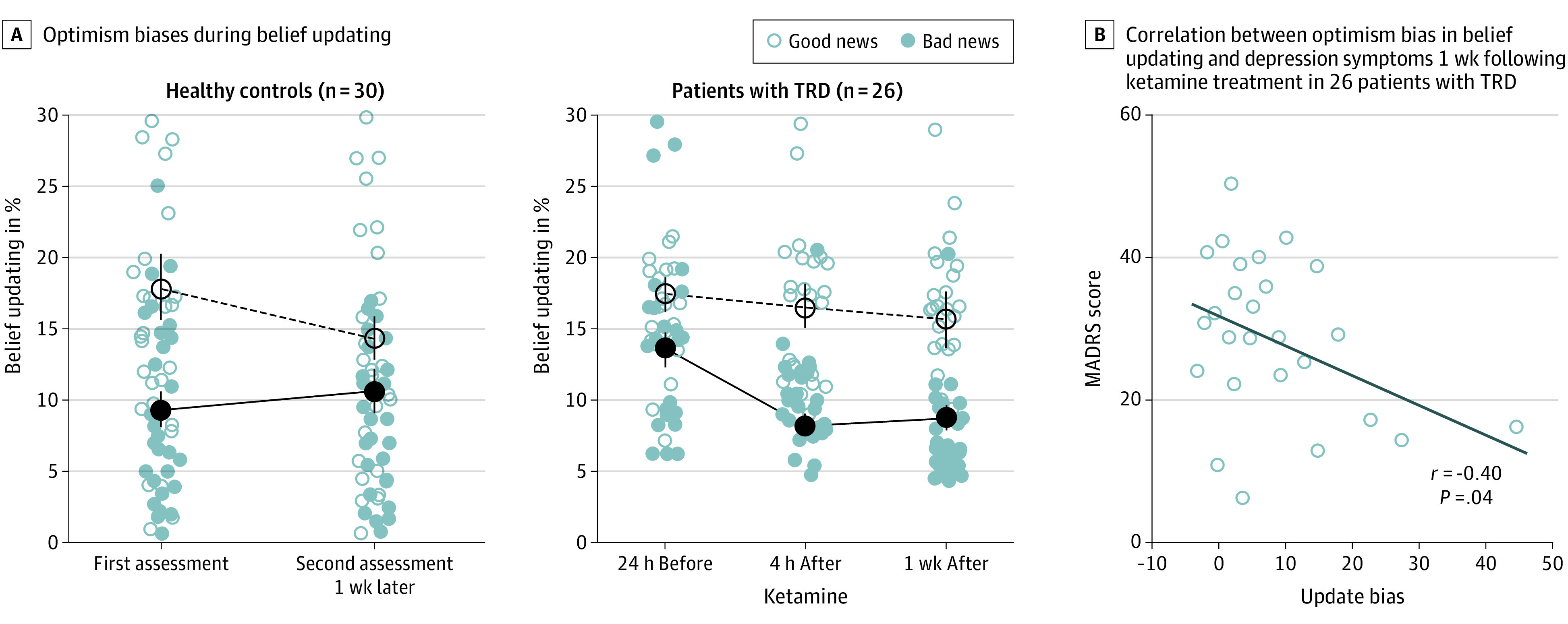
Behavioral Results for 26 Patients With Treatment-Resistant Depression (TRD) and 30 Healthy Controls A, The line graphs show the observed average belief updating after favorable (good) and unfavorable (bad) news. Belief updating corresponds to the absolute difference between the first and second estimate for good and bad news trials, respectively. Error bars correspond to the SE of the mean. B, Correlation between the depression score and belief updating for 26 patients with TRD after 1 week of ketamine treatment. The scatterplot displays the covariance of global clinical symptoms of depression (mean Montgomery-Åsberg Depression Rating Scale score) and good news/bad news updating bias.

A linear mixed-effects model of belief updating detected a significant 3-way interaction of group by testing time by EE valence (β = −0.91; 95% CI, −1.58 to −0.24; *t*_216_ = −2.67; *P* = .008; eTable 6 in the Supplement). As soon as 4 hours after the first ketamine infusion, patients with TRD updated their beliefs more often after good than after bad news relative to baseline and sequential testing in healthy controls (Figure 3A). Similar effects were obtained when comparing the effect of EE valence on belief updating at baseline to one week after ketamine treatment (group × testing time × EE valence interaction: β = −0.73; 95% CI, −1.42 to −0.04; *t*_216_ = −2.1; *P* = .03; eTable 7 in the Supplement).

The results were specific to belief-updating biases. The interaction group × testing time × EE valence was nonsignificant for initial belief estimates (eTable 8 in the Supplement), personal relevance of events (eTable 9 in the Supplement), EE magnitude (eTable 10 in the Supplement), and confidence in the base rates (eMethods 5 and eTable 11 in the Supplement).

The emergence of the optimism bias in belief updating correlated negatively with the depressive symptoms measured by the MADRS score after 1 week of treatment. Patients who displayed stronger optimism biases in belief updating were also those who displayed fewer depressive symptoms (*r* = −0.4; *P* = .04; permuted chance correlations 95% CI, −0.32 to 0.33; Figure 3B).

### Computational Modeling Results of the Emergence of Optimism Biases in Belief Updating

**Figure 4.**
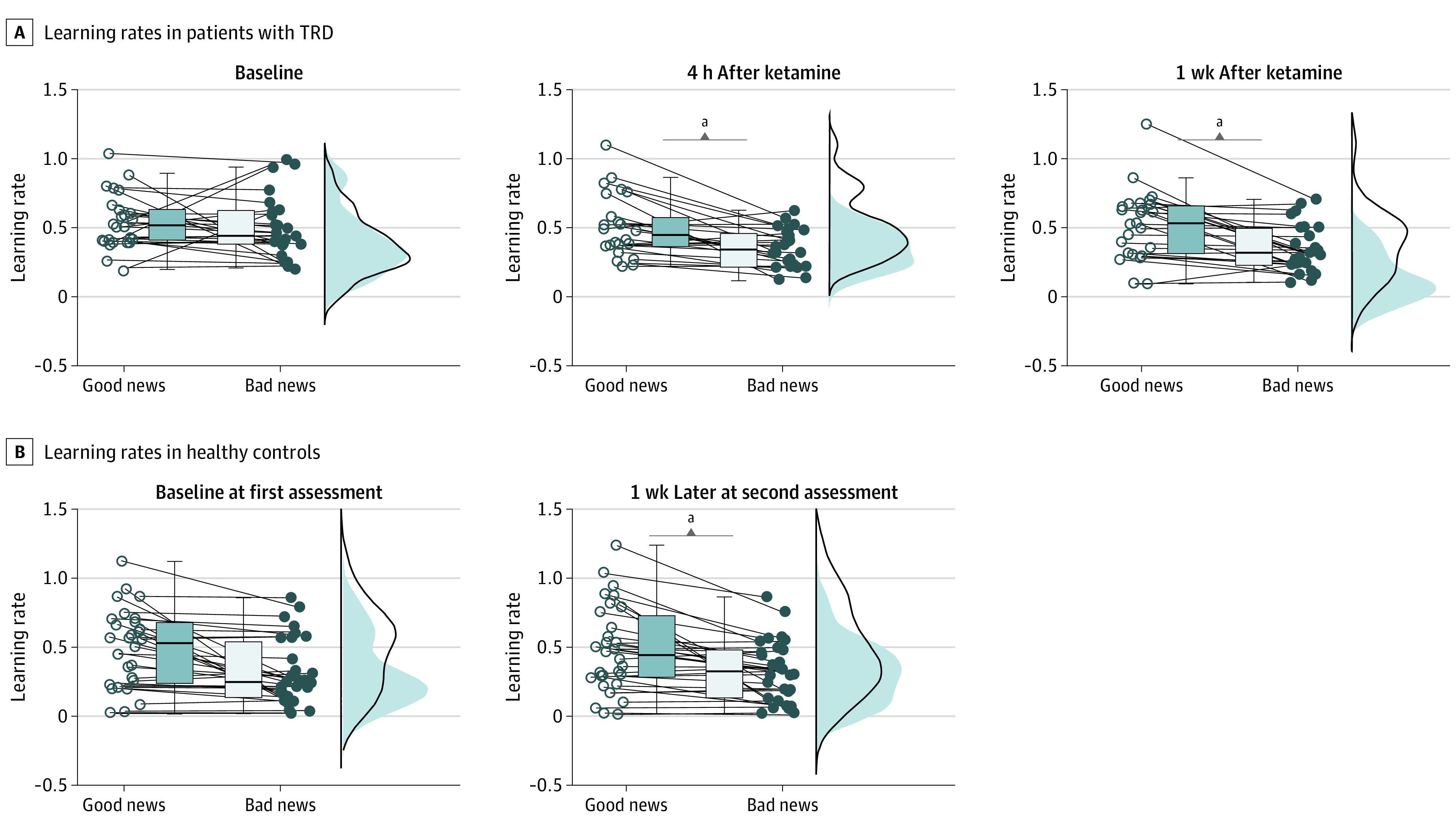
Model-Based Results Boxplot graphs showing the IQRs for learning rates obtained from fitting a generic reinforcement learning model to the observed belief updates in 26 patients with treatment-resistant depression (TRD) (A) and 30 healthy control participants (B). The jitter element to the side of each boxplot displays individual learning rates, with each dot representing 1 patient. ^a^*P* < .05.

Linear mixed-effects modeling of LRs at baseline showed a significant interaction of group × EE valence (β = 0.03; 95% CI, 0-0.06; *t*_106_ = 2.03; *P* = .04; eTable 14 in the Supplement), which indicated that patients with TRD learned similarly from positive and negative EEs before ketamine treatment (T0: good news LR, 0.53 [SEM, 0.04]; bad news LR, 0.49 [SEM, 0.04]; *t*_25_ = 0.68; *P* = 59). After ketamine treatment, this interaction became nonsignificant (β = 0.002; 95% CI, −0.03 to 0.03; *t*_106_ = 0.12; *P* = .90; eTables 15 and 16 in the Supplement). Post hoc *t* tests indicated that LRs from positive EEs were greater than the those from negative EEs (T1: good news LR, 0.51 [SEM, 0.04]; bad news LR, 0.36 [SEM, 0.03]; *t*_25_ = 3.8; *P* < .001; T2: good news LR, 0.53 [SEM, 0.05]; bad news LR, 0.37 [SEM, 0.03]; *t*_25_ = 4.1; *P* < .001; Figure 4A).

Further explorations of the drug effect on LRs revealed that it was driven by a significant difference in the LRs from negative EEs between baseline and 4 hours after the first single ketamine infusion (bad news LR : T0, 0.49 [SEM, 0.04]; T1, 0.36 [SEM, 0.03]; *t*_25_ = 2.5; *P* = .02).

### Mediation Analysis of Optimism Bias in Belief Updating and Effect of Ketamine on Clinical Improvement

**Figure 5.**
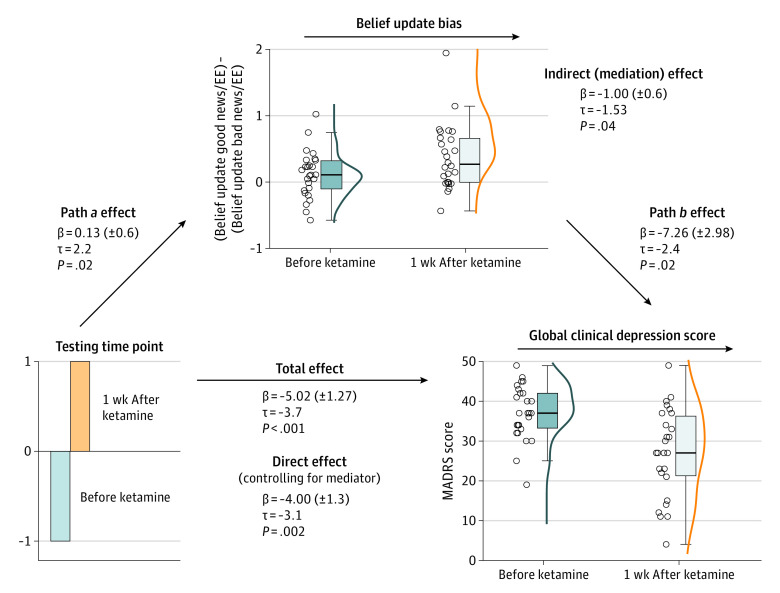
Mediation Results Boxplots showing the IQRs for belief-updating biases (normalized by estimation error magnitude) and Montgomery-Åsberg Depression Rating Scale scores 24 h before and 1 wk after ketamine treatment. The black lines inside the boxplots show the median values, and the jitter elements left of each boxplot show the individual patients. EE indicates estimation error.

Mediation analysis found a significant path *a* regression that replicated the main effect of ketamine on belief-updating biases in patients with TRD (β = 0.13; *t* = 2.21; *z* = 2.39; *P* = .02; Figure 5). Optimistically biased belief updating also predicted lower MADRS scores, controlling for the main effect of ketamine on belief-updating biases (path *b* regression: β = −7.26; *t* = −2.43; *z* = −2.32; *P* = .02). Finally, the direct effect of ketamine on MADRS scores was significantly reduced when controlling for belief-updating biases (direct effect, path *c′*, after controlling for the mediator: β = −4.00; *t* = −3.11; *z* = −2.89; *P* = .004; total effect, path *c*: β = −5.00; *t* = −3.95; *z* = −3.69; *P* < .001), indicating a partial mediation effect (indirect effect, path a*b: β = −1.00; *t* = −1.53; *z* = −1.98; *P* = .04).

## Discussion

The scope of this observational study was to measure belief-updating mechanisms in a rare cohort of patients with TRD who were tested in their usual measurement-based care setting before and after receiving needed ketamine treatment.^31^ The results showed, in line with previous studies,^24,25^ that ketamine induced a rapid and sustained decrease in depressive symptoms. On the cognitive level, patients with TRD showed weaker optimism biases in belief updating at baseline compared with 4 hours after the first ketamine infusion. The significant increase in the optimism bias correlated with the reduction in MADRS scores after 1 week of treatment and formally mediated the clinical effect of ketamine at 1 week of treatment. Computational analyses showed that this effect was underpinned by asymmetrically increased learning from positive rather than negative EEs.

MDD is associated with negative expectations and decreased sensitivity to expectation violation.^2,17,20,22,44,45^ Our finding of an attenuated optimistic bias in belief updating prior to ketamine treatment in patients with TRD is consistent with those of several studies that have used the same belief-updating task on patients with MDD^17,18^ and others that have validated it for detecting belief-updating biases in healthy participants,^11,46,47^ including test-retest situations.^41,48,49^ Importantly, to our knowledge, our study is the first to show attenuated optimistic biases in patients with TRD before compared with after treatment. We found that the sensitivity of patients with TRD to negative EEs decreased after ketamine treatment, whereas the sensitivity to positive EEs remained similar across testing time points. This finding is at odds with results reported by Korn et al^17^ and Kube et al,^44^ who showed that MDD was associated with less sensitivity to positive disconfirmation. Several factors may explain these discrepancies, including the task itself and its cognitive demands as well as its content and the clinical heterogeneity of patients with depression. However, in accordance with our findings, Garrett et al^18^ reported that patients with depression were more sensitive to bad news than good news and updated their beliefs more after a negative EE. Importantly, this previous study found that the lack of optimism bias correlated with the severity of depression.^17,18^ Here, we provide observational and novel evidence that this correlation was reversed following ketamine treatment.

Our model-based findings are consistent with the observed effects and provide insight into the learning mechanisms of belief updating in patients with TRD, which could be due to a learning deficit. However, when decomposing the LRs, we found that ketamine triggered a change in the asymmetry component, which indicated how much participants learned from positive rather than negative EEs. On the contrary, the alpha parameter, which indicated how much participants updated after an EE, did not differ between testing time points or participant groups. These findings suggest that the differences in the LRs were due to the emergence of valence-biased learning and not an absolute deficit to learn from EEs. 

### Limitations

This study has limitations. The age-matched and education-matched cohort of healthy control participants were included to alleviate concerns about sequential testing effects. None of the healthy control participants presented with depressive symptoms, but the study cannot infer specificity of findings to ketamine effects in patients with TRD. It may well be that ketamine has similar effects on optimistically biased belief updating in healthy controls. However, this question goes beyond the scope of our study. Ketamine in healthy controls is used as a pharmacological model of early psychosis to perturb brain mechanisms that reduce uncertainty and how EEs are used to update beliefs.^29,50^ More studies are needed to disentangle the specific effects of ketamine on belief updating and belief-updating biases in different participant cohorts. In this study, ketamine was added as a strategy to achieve stronger and more sustained antidepressant responses, and patients received ketamine in addition to their ongoing antidepressant treatment. It is possible that different types of antidepressant treatment strategies also modulate belief updating. Moreover, it may be that other cognitive processes, such as emotion processing are affected as well. For example, prior work has focused on the mechanisms of clinical improvement following monoaminergic antidepressants, such as selective serotonin reuptake inhibitors.^51,52^ Findings from this line of research showed that early responses to serotonin reuptake inhibitor treatment involve a reduction in negative biases in emotion processing and potentially give rise to more slowly evolving improvements in mood and depressive symptoms. We call for more research to shed light onto the neurocognitive processes through which different antidepressant treatment strategies produce clinical improvement.

## Conclusions

In conclusion, our results converge toward the finding that ketamine improves TRD and that this improvement is associated with changes in the belief-updating processes underpinned by increased asymmetric learning from positive rather than negative EEs. These findings shed light on the possible mechanisms underlying ketamine’s rapid antidepressant effects and pave the way toward the use of ketamine-augmented psychotherapy protocols.

## Notes

Supplement. **eMethods 1.** Ketamine Administration **eMethods 2.** Fine-Grained Analysis of Clinical Improvements on Subscores of the MADRS **eMethods 3.** Correlation Between Patients’ Anticipation of Treatment Outcomes and MADRS Scores **eMethods 4.** Dependent Variables of the Belief-Updating Task **eMethods 5.** Supplementary Results of the Belief-Updating Task **eMethods 6.** Results With Paradoxical Trials **eMethods 7.** Reinforcement Learning Model **eMethods 8.** Details of the Mediation Model **eMethods 9.** Task Instructions in French **eTable 1.** Demographics **eTable 2.** Clinical Characteristics of TRD Patients (n = 26) **eTable 3.** Improvement in Depression Measured by the MADRS Before and After Ketamine Treatment for 26 TRD Patients **eTable 4.** Average Number of Trials Excluded From Analyses of the Belief-Updating Task **eTable 5.** Belief-Updating Task Variables **eTable 6.** Linear Mixed-Effects Model Results Fitting the Average Absolute Belief Updates (UPD) Before (T0) and 4 h After the First Ketamine Infusion (T1) **eTable 7.** Linear Mixed-Effects Model Results Fitting the Average Absolute Belief Updates (UPD) Before (T0) and 1 Week After the First Ketamine Infusion (T2) **eTable 8.** Linear Mixed-Effects Model Results Fitting the Average Initial Belief Estimates (E1) at Baseline and 4 h After Ketamine Treatment **eTable 9.** Linear Mixed-Effects Model Results Fitting the Personal Relevance of Events At Baseline and 4 h After Ketamine Treatment **eTable 10.** Linear Mixed-Effects Model Results Fitting Absolute Estimation Error Magnitude at Baseline and 4 h After Ketamine Treatment **eTable 11.** Linear Mixed-Effects Model Results Fitting Confidence Ratings in the Base Rates of Events at Baseline and 4 h After Ketamine Treatment **eTable 12.** Linear Mixed-Effects Model Results Fitting the Update Bias at Baseline and 4 h After Ketamine Treatment **eTable 13.** Linear Mixed-Effects Model Results Fitting Belief Updates, Including Paradoxical Trials, at Baseline and 4 h After Ketamine Treatment **eTable 14.** Linear Mixed-Effects Model Results Fitting Learning Rates at Baseline **eTable 15.** Linear Mixed-Effects Model Results Fitting Learning Rates 4 h After the First Ketamine Infusion **eTable 16.** Linear Mixed-Effects Model Results Fitting Learning Rates 1 Week After the First Ketamine Infusion **eTable 17.** Model Comparisons TRD Patients **eTable 18.** Model Comparisons Healthy Controls
eReferences.
