# Supplementary material for: Evaluation of Early Ketamine Effects on Belief-Updating Biases in Patients With Treatment-Resistant Depression
Source: JAMA Psychiatry. 2022 Sep 28;79(11):1124–32. doi: 10.1001/jamapsychiatry.2022.2996 (PMC9520441; doi:10.1001/jamapsychiatry.2022.2996)
Supplement: Supplement. — eMethods 1. Ketamine Administration eMethods 2. Fine-Grained Analysis of Clinical Improvements on Subscores of the MADRS eMethods 3. Correlation Between Patients’ Anticipation of Treatment Outcomes and MADRS Scores eMethods 4. Dependent Variables of the Belief-Updating Task eMethods 5. Supplementary Results of the Belief-Updating Task eMethods 6. Results With Paradoxical Trials eMethods 7. Reinforcement Learning Model eMethods 8. Details of the Mediation Model eMethods 9. Task Instructions in French eTable 1. Demographics eTable 2. Clinical Characteristics of TRD Patients (n = 26) eTable 3. Improvement in Depression Measured by the MADRS Before and After Ketamine Treatment for 26 TRD Patients eTable 4. Average Number of Trials Excluded From Analyses of the Belief-Updating Task eTable 5. Belief-Updating Task Variables eTable 6. Linear Mixed-Effects Model Results Fitting the Average Absolute Belief Updates (UPD) Before (T0) and 4 h After the First Ketamine Infusion (T1) eTable 7. Linear Mixed-Effects Model Results Fitting the Average Absolute Belief Updates (UPD) Before (T0) and 1 Week After the First Ketamine Infusion (T2) eTable 8. Linear Mixed-Effects Model Results Fitting the Average Initial Belief Estimates (E1) at Baseline and 4 h After Ketamine Treatment eTable 9. Linear Mixed-Effects Model Results Fitting the Personal Relevance of Events At Baseline and 4 h After Ketamine Treatment eTable 10. Linear Mixed-Effects Model Results Fitting Absolute Estimation Error Magnitude at Baseline and 4 h After Ketamine Treatment eTable 11. Linear Mixed-Effects Model Results Fitting Confidence Ratings in the Base Rates of Events at Baseline and 4 h After Ketamine Treatment eTable 12. Linear Mixed-Effects Model Results Fitting the Update Bias at Baseline and 4 h After Ketamine Treatment eTable 13. Linear Mixed-Effects Model Results Fitting Belief Updates, Including Paradoxical Trials, at Baseline and 4 h After Ketamine Treatment eTable 14. Linear Mixed-Effects Model Results Fi [file jamapsychiatry-e222996-s001.pdf]

Supplemental Online Content

Bottemanne H, Morlaas O, Claret A, Sharot T, Fossati P, Schmidt L. Evaluation of early ketamine effects on belief-updating biases in patients with treatment-resistant depression. *JAMA Psychiatry*. Published online September 28, 2022. doi:10.1001/jamapsychiatry.2022.2996

eMethods 1. Ketamine Administration

eMethods 2. Fine-Grained Analysis of Clinical Improvements on Subscores of the MADRS

eMethods 3. Correlation Between Patients' Anticipation of Treatment Outcomes and MADRS Scores

eMethods 4. Dependent Variables of the Belief-Updating Task

eMethods 5. Supplementary Results of the Belief-Updating Task

eMethods 6. Results With Paradoxical Trials

eMethods 7. Reinforcement Learning Model

eMethods 8. Details of the Mediation Model

eMethods 9. Task Instructions in French

eTable 1. Demographics

eTable 2. Clinical Characteristics of TRD Patients (n = 26)

eTable 3. Improvement in Depression Measured by the MADRS Before and After Ketamine Treatment for 26 TRD Patients

eTable 4. Average Number of Trials Excluded From Analyses of the Belief-Updating Task

eTable 5. Belief-Updating Task Variables

eTable 6. Linear Mixed-Effects Model Results Fitting the Average Absolute Belief Updates (UPD) Before (T0) and 4 h After the First Ketamine Infusion (T1)

eTable 7. Linear Mixed-Effects Model Results Fitting the Average Absolute Belief Updates (UPD) Before (T0) and 1 Week After the First Ketamine Infusion (T2)

eTable 8. Linear Mixed-Effects Model Results Fitting the Average Initial Belief Estimates (E1) at Baseline and 4 h After Ketamine Treatment

© 2022 Bottemanne H et al. *JAMA Psychiatry*.

eTable 9. Linear Mixed-Effects Model Results Fitting the Personal Relevance of Events At Baseline and 4 h After Ketamine Treatment

eTable 10. Linear Mixed-Effects Model Results Fitting Absolute Estimation Error Magnitude at Baseline and 4 h After Ketamine Treatment

eTable 11. Linear Mixed-Effects Model Results Fitting Confidence Ratings in the Base Rates of Events at Baseline and 4 h After Ketamine Treatment

eTable 12. Linear Mixed-Effects Model Results Fitting the Update Bias at Baseline and 4 h After Ketamine Treatment

eTable 13. Linear Mixed-Effects Model Results Fitting Belief Updates, Including Paradoxical Trials, at Baseline and 4 h After Ketamine Treatment

eTable 14. Linear Mixed-Effects Model Results Fitting Learning Rates at Baseline

eTable 15. Linear Mixed-Effects Model Results Fitting Learning Rates 4 h After the First Ketamine Infusion

eTable 16. Linear Mixed-Effects Model Results Fitting Learning Rates 1 Week After the First Ketamine Infusion

eTable 17. Model Comparisons TRD Patients

eTable 18. Model Comparisons Healthy Controls

eReferences.

This supplementary material has been provided by the authors to give readers additional information about their work.

© 2022 Bottemanne H et al. *JAMA Psychiatry*.

eMethods 1. Ketamine Administration

Subanesthetic doses of ketamine (ketamine-ratiopharm, 500 mg/10 mL, Ratiopharm) were infused for 40 min (0.5 mg/kg) intravenously. In total, all patients received three infusions over one week, with 48 h between the first two infusions and 72 h between the last two infusions. After each infusion, patients were discharged home following a 4-h recovery period.

© 2022 Bottemanne H et al. *JAMA Psychiatry*.

eMethods 2. Fine-Grained Analysis of Clinical Improvements on Subscores of the MADRS

Although we observed a rapid improvement in depression, the clinical response rate following ketamine appeared to be very low relative to previous, open-labeled studies [1,2]. This could be partially related to the timing of the assessments, which were completed 4 h after the first and third infusions. The existing literature is relatively consistent in showing that the peak responder rate is generally observed at 24 h following a single infusion[1] and the timing of assessments we used may have been too close to the infusions. Moreover, we included patients with high levels of disease severity (mean MADRS: 37.1 ± 1.4) and treatment resistance (mean MSM: 9.4 ± 0.4). Among our patients, 77% had severe depression according to MADRS criteria (score > 34), and the average duration of the current episode was 5.7 years (± 0.8), which is a long duration. Even more striking, 46% had severe treatment resistance and 54% moderate treatment resistance according to MSM criteria (score > 11 and score > 7, respectively). The degree of severity and resistance to treatment could explain the low level of clinical response after only three ketamine infusions. More studies are needed to assess belief updating after more than three ketamine infusions and over a greater time span.

Based on the study of Quilty et al. [3], MADRS sub-scores were calculated based on the four factors that have been reported to be stable across time and gender: sadness (items 1 and 2), neurovegetative symptoms (items: inner tension, sleep, and appetite), detachment (items: feeling of incapacity, concentration, and lassitude), and negative thoughts (items: pessimism and suicidal ideas). Signed Wilcoxon rank tests revealed that early treatment responses, measured 4 h after the first infusion, involved

© 2022 Bottemanne H et al. *JAMA Psychiatry.*

improvements in sadness, negative thoughts, and neurovegetative symptoms. Improvement in detachment evolved more slowly and was significant at one week after the first infusion (eTable 3).

© 2022 Bottemanne H et al. *JAMA Psychiatry*.

eMethods 3. Correlation Between Patients' Anticipation of Treatment Outcomes and MADRS Scores

Meta-analyses of pharmacological antidepressant treatment have shown that patients who expect to benefit from treatment respond best to antidepressant medication [4]. This relationship between pharmacological effects and beliefs has led to the response expectancy theory of placebo effects [5]. To assess the extent to which patients anticipated positive treatment outcomes, expectancy ratings were collected at T0, 24 h prior to the first ketamine infusion. Thus, patients rated the following types of expectancies on a visual analogue scale between 0 and 100: (A) expected drug efficiency (i.e. how much they expect the treatment to be efficient within the next few days), (B) expected response (i.e. how intense they expect their depression to be after treatment with ketamine), and (C) expectation of remission (i.e. how much they expect remission after treatment). Due to technical problems, these ratings were obtained for only 18 of the 26 TRD patients.

We explored the idea that the patients who believe they will get better are also those who benefit most from treatment by assessing how much of a patient's anticipation in a positive treatment outcome moderated their global clinical improvement after ketamine treatment. Thus, three Pearson's correlations were conducted between global clinical improvement (i.e., expressed by the difference in the MADRS score before and one week after ketamine treatment), and (A) expected response, (B) expected drug efficiency, and (C) expectation of remission ratings that were measured 24 h before the first ketamine infusion at T0. The threshold for statistical significance was $p = 0.016$, corresponding to a Bonferroni correction for multiple comparisons.

We found a significant positive correlation between expected drug efficiency and global clinical improvement at one week after treatment relative to baseline ($r = 0.63$, $p = 0.004$, eFigure 1A). Moreover, patients who expected less intense depressive symptoms after treatment also showed fewer depressive symptoms after one week relative to baseline ($r = -0.58$, $p = 0.01$, eFigure 1B). Expected remission from depression and global clinical improvement trended toward a positive correlation ($r = 0.28$, $p = 0.25$).

The positive correlation between anticipated treatment outcome and clinical improvement is in accordance with the results of a previous studies [4]. These patients have often experienced the failure of many different lines of treatment and often perceive ketamine as the last chance for a cure. Here, patients were addressed to our mood center after the failure of at least two antidepressants, which might have induced the hope to benefit from a new treatment strategy, such as ketamine. At the time of the study, ketamine was a novel treatment strategy for depression and was implemented in only a handful of clinical departments in France. This context may have induced anticipation of positive treatment outcomes by patients and may explain their beneficial role in treatment responsiveness [4,5]. Of note, these two types of expectation (i.e., prognostic response and drug efficiency) did not significantly correlate with the emergence of optimism biases in belief updating. This finding suggests that changes in expectations improve depressive symptoms via two pathways: belief-updating bias and prognostic response and expected drug efficiency.

© 2022 Bottemanne H et al. *JAMA Psychiatry.*

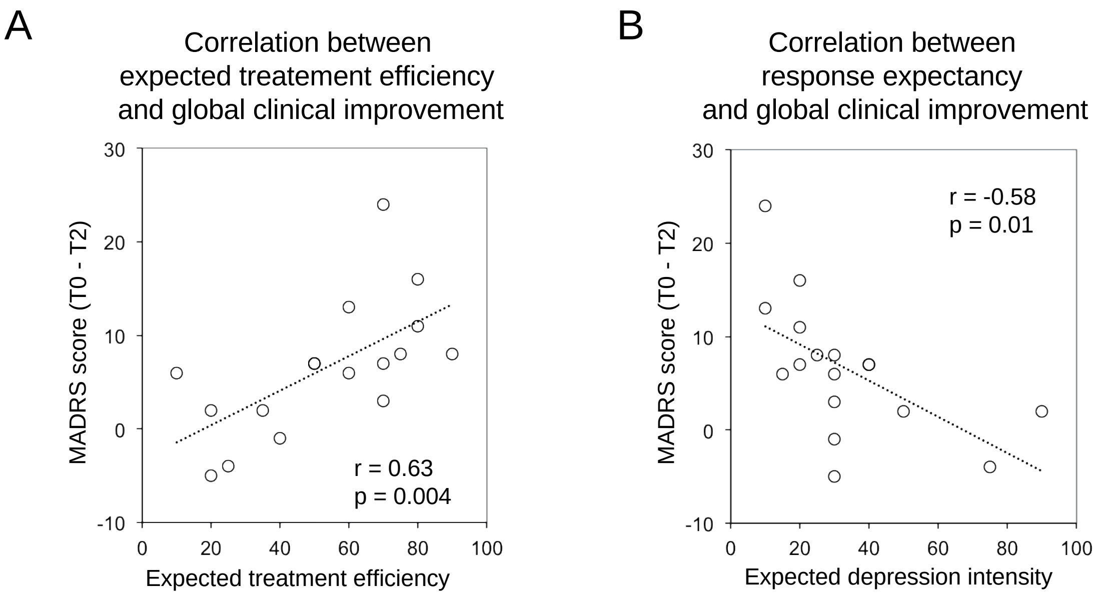

eFigure 1. Scatterplots showing, for N = 18 TRD patients, correlations between global clinical improvements from baseline (T0) to 4 h after the third ketamine infusion at one week of treatment (T2) and **(A)** expected treatment efficiency and **(B)** expected response (i.e. response expectancy). Each dot corresponds to one TRD patient.

© 2022 Bottemanne H et al. *JAMA Psychiatry*.

eMethods 4. Dependent Variables of the Belief-Updating Task

In total, 120 adverse life events were used and randomly allotted to three lists of 40 trials. The actual base rates for 60 of the 120 events were taken from previously published studies on healthy and depressed patients[6,7]. The remaining 60 events and their base rates were newly created for this study. Base rates ranged between 10 and 70%. To ensure that the range of possible overestimation was equal to the range of possible underestimation, participants estimated their beliefs anywhere within the range of 5 and 95%.

Participants used the numerical buttons on the computer keyboard to enter their responses and pressed the space key to confirm each response. All responses were self-paced, and participants were required to respond to go to the next rating and trial. All subjects completed a practice session of three trials before beginning the main experiment. The task was identical at all testing timepoints, except for the fact that a different set of adverse life events was used in each testing session. Thus, the 120 life events were randomly divided into three lists of 40 different adverse life events, with one list for each timepoint (T0, T1, T2). The order of the lists was counterbalanced across participants (Latin square design).

The task measured the dependent variable – belief updating (UPD), which reflected the percentage to which participants changed their initial belief estimate (E1) relative to their second belief estimate (E2) given after being provided with information about the actual base rates of experiencing a given adverse life event, according to equation 1:

$$(1)\ UPD = E1 - E2$$

© 2022 Bottemanne H et al. *JAMA Psychiatry.*

Belief updating (UPD) was driven by the magnitude of the estimation errors (EEs), which indicated whether participants initially overestimated or underestimated their actual likelihood of experiencing an adverse life event (E1), relative to its actual base rate (aBR), and was calculated according to equation 2:

$$(2)\ EE = E1 - aBR$$

The estimation error was further used to categorize trials into good or bad news trials: for good news trials, the estimation error was positive ($EE > 0$), which indicated an initial overestimation of one's likelihood of experiencing an adverse life event relative to the actual base rate of that event ($E1 > aBR$). For bad news trials, the estimation error was negative ($EE < 0$), which indicated an initial underestimation of one's likelihood of experiencing an adverse life event relative to its actual base rate of occurrence ($E1 < aBR$).

The following additional variables were calculated with the participant responses during the belief-updating task:

<u>The updating bias:</u>

A complementary way to test belief-updating biases (UDB) is to directly calculate the update bias according to equation 3:

$$(3)\ UDB = |UPD|_{\text{good news}} - |UPD|_{\text{bad news}}$$

A positive difference indicates that participants updated beliefs about their lifetime risks of experiencing adverse life events to a greater extent after good news than after bad news. Furthermore, belief updating is driven by the magnitude of estimation errors. To remove this confounder, the absolute updates for each participant were first averaged for good and bad news trials and then divided by the average absolute estimation error ($|EE|$) in good news and bad news trials. The updating bias (UDP) normalized by the magnitude of EE was then calculated according to equation 4:

© 2022 Bottemanne H et al. *JAMA Psychiatry.*

$$(4)\ UDB = (|UPD| / |EE|)_{good\ news} - (|UPD| / |EE|)_{bad\ news}$$

A positive difference indicates that participants updated beliefs about their lifetime risks of experiencing adverse life events to a greater extent after good news than after bad news. To note, trials with zero estimation error and non-responses were excluded from these calculations.

The general knowledge about lifetime risks, called the distance: This variable expresses how much participants consider their own likelihood of experiencing a given adverse event to be different from the lifetime risk of a person with a similar socio-economic background. It is calculated according to equation 5:

$$(5)\ distance = eBR - E1$$

Moreover, this difference and the estimation error were used to calculate the personal relevance of a life event following equations 6 and 7:

$$(6)\ PR = 1 - (distance / (eBR - 1))\ \textit{for trials when } E1 < eBR$$

$$(7)\ PR = 1 - ((1 - distance)/(99 - eBR))\ \textit{for trials when } E1 > eBR$$

This measure corresponds to the concept of 'relative personal relevance', as described by Kuzmanovic and Rigoux 2017 [8]. Equations (5) and (6) formalize PR as a score between 1 and 0, with 1 indicating equal risk perception (e.g. minimal difference or relative PR) for oneself and someone else. A relative PR = 0 indicates that the participant's own risk is maximally different (or minimally equal) from the risk for someone else. Note the PR score expresses personal relevance relative to the average, irrespective of whether participants over- or underestimated their risk. For example, if a participant displayed a risk perception of 90% for a given event that was very different relative to someone else's risk (10%), the distance measure is negative and high (e.g.

© 2022 Bottemanne H et al. *JAMA Psychiatry.*

distance = -80% = 10 - 90). Equation (5) then gives a relative PR value of 0.089, which is close to 0 and indicates minimal matching (maximal difference) of the risk perception for oneself and someone else and irrespective of the valence of this relative risk perception (i.e., whether it is a negative, over-, or positive underestimation).

Similarly, the PR value is close to zero if the initial estimation of the participant was extremely low (distance = 80 = 90 - 10, 10%) relative to someone else's risk (90%). In this case, equation (6) gives the same PR value of 0.089, indicating maximal relative relevance (or minimal matching of E1 and eBR). On the contrary, if a participant perceives his/her risk to be average relative to someone else's (e.g. distance = 40 – 30 = 10), the PR score will be close to 1 (e.g., 0.74). This score then, irrespective of the valence of the distance measure, indicates personal relevance equal to that, on average, in society.

The original RL-like model of belief updating validated by Kuzmanovic and Rigoux included personal relevance as a weighting factor of the influence of estimation error on belief updating. However, in our data, we found that a simpler model without personal relevance best explained the observed belief-updating behaviour of participants (see model comparisons). That is why we solely explored group and testing timepoint effects on personal relevance reported in eTable 10.

Psychometric properties of the belief-updating task: Test-retest reliability

We performed a test-retest correlational analysis for both healthy controls and TRD patients to check for the psychometric properties of the belief-updating task to reliably assess belief-updating biases. Specifically, Pearson's correlation coefficients tested whether the participants who presented a good news/bad news belief-updating bias,

© 2022 Bottemanne H et al. *JAMA Psychiatry*.

controlled for estimation error magnitude at baseline, also presented the bias at later testing timepoints. However, we also expected this correlation to be smaller in the TRD patients when comparing the baseline to post-ketamine treatment testing timepoints, because the patients were expected to show a weak bias at baseline consistent with previous studies on depressed patients [4]. On the contrary, given our hypothesis about the effects of ketamine, the correlation should become significant, similar to that of the healthy controls, following ketamine treatment.

Consistent with these hypotheses, belief-updating biases at T0 and T1 correlated positively and significantly for healthy volunteers (Pearson's $r = 0.47$, $p = 0.0075$). For the TRD patients the belief-updating biases at the two post-ketamine treatment testing timepoints also correlated significantly ($r = 0.4$ $p = 0.05$; $r = 0.6$, $p = 0.0004$). However, as expected and consisted with the main findings, the weak belief-updating biases at baseline did not correlate with the much stronger belief-updating biases measured at the two testing timepoints following ketamine treatment (T0 to T1: $r = 0.1$, $p = 0.6$; T0 to T2: $r = 0.3$, $p = 0.2$).

© 2022 Bottemanne H et al. *JAMA Psychiatry*.

eMethods 5. Supplementary Results of the Belief-Updating Task

The linear mixed effects model fitted to belief updating further showed that participants updated their beliefs less the more often they performed the task (ß = -0.78, t(216) = -2.2, p = 0.02, 95%CI [-1.4 ; -0.1]) and more after a positive effect on estimation error valence (ß = 3.02, t(216) =6.7, p = 1.4e-10, 95%CI [2.1 ; 3.9]), eTables 6 and 7). No other effects were significant.

Group and testing time effects on other variables of belief updating

To test for potential differences across testing timepoints, good and bad news trials, and groups, the following belief-updating variables were fitted by linear mixed effects models according to equation 8:

(8) ***DV = 1 + Group + Time + Valence + |EE|* + age + education + Group*Time*Valence + (1|subject) + (1+EEvalence|subject) + (1+|EE||subject)****

This linear mixed effect model was fitted to the following dependent variables (DV): personal relevance of events, absolute estimation error magnitude, and confidence in the base rate.

The model included the following fixed effects: estimation error valence (coded -1 for bad news and 1 for good news), group (coded 1 for healthy controls and -1 for TRD patients), testing timepoint (coded -1 for baseline and 1 for 4 h after a first ketamine infusion for the TRD patients or 2nd assessment for healthy controls), absolute estimation error magnitude (|EE|) (* except for models fitting the estimation error magnitude as a dependent variable (DV)), age, and level of education. The models further included fixed effects for the three-way interaction ‘group by time by valence’ to test whether the effects changed as a function of whether participants over- or underestimated their likelihood, (i.e., estimation error valence), sequential testing (i.e.,

© 2022 Bottemanne H et al. *JAMA Psychiatry*.

testing timepoint), and participant group (i.e., TRD versus healthy controls). The model also nested the intercept by participant number to control for inter-individual differences in the DVs at the random level and random slopes for the effect of estimation error valence and magnitude.

Differences in initial belief estimates between groups and testing timepoints and good/bad news trials

We checked whether the ketamine effect observed on belief updating was driven by differences between TRD patients and controls in the initial belief estimates by conducting a linear mixed effects model according to equation (9):

(9) **E1 ~ 1 + group + time + EEvalence + age + education + group:time:EEvalence + (1+EEvalence | subject) + (1+|EE| | subject)**

The results are reported in SI Table 8 and show that the effects of ketamine cannot be explained by differences in the first estimate between TRD patients and healthy controls, as indicated by a non-significant three way interaction group by time by estimation error valence for the initial beliefs estimates (E1 T0 vs T1: ß = -0.75, t(216) = -1.68, p = 0.09, 95%CI [-1.6 ; 0.12], eTable 8).

Differences in the personal relevance of events between groups, testing timepoints good/bad news trials

The model detected a main effect of group (ß = 0.03, t(216) = 2.3, p = 0.02, 95%CI [0.004;0.05]), estimation error valence (ß = 0.03, t(216) = 3.7, p = 0.0002, 95%CI [0.02;0.05]), and absolute estimation error magnitude (ß = -0.004, t(216) = -2.7, p = 0.007, 95%CI [-0.007;-0.001]), without any significant interaction group by time by EE valence (eTable 10).

© 2022 Bottemanne H et al. *JAMA Psychiatry*.

Differences in estimation error magnitude between groups, testing timepoints, and good/bad news trials

The model detected a main effect of testing timepoint (ß = -1.13, t(217) = -3.5, p = 0.0006, 95%CI [-1.7; -0.49]). The estimation error magnitude decreased the more often participants performed the task. No other main effects of group, estimation error valence, or interactions were significant, which suggests that the magnitude of estimation errors did not differ between participant groups (TRD patients vs healthy controls) or good and bad news trials (eTable 11).

Differences in confidence in the base rates between groups, testing timepoints, and good/bad news trials

The model detected no significant main effects or interactions on confidence in the base rate (eTable 12).

© 2022 Bottemanne H et al. *JAMA Psychiatry*.

eMethods 6. Results With Paradoxical Trials

For the main analyses reported in the main manuscript, paradoxical trials were excluded from the update measure. These were trials in which participant estimates increased despite good news and decreased despite bad news and, thus, the responses were removed from analyses. It is not clear how to interpret these trials. They could be error trials due to fatigue or a confirmation bias. We included a particularly symptomatic population (high MADRS and high resistance score) and the experimenters observed significant cognitive fatigability in the patients.

The results still hold when keeping these trials in a linear mixed effects model, analogous to that reported in the methods section of the main manuscript, was fit to update measures with the paradoxical trials kept in. The three-way interaction 'time by group by EE valence' was still significant when comparing belief updates at baseline and 4 h after ketamine (ß = -0.79, t(216) = -2.05, p = 0.04, 95%CI [-1.5; -0.03], eTable 13.

© 2022 Bottemanne H et al. *JAMA Psychiatry*.

eMethods 7. Reinforcement Learning Model

The model relies on a generic reinforcement learning (RL) algorithm that assumes belief updating to be proportional to the size of the EEs, which are themselves weighted by the learning rate (LR) following equation 10:

(10) Belief update = LR * EE

The learning rate determines how much beliefs are updated as a function of the size of the EEs. Consistent with previous work and to test for the good news/bad news bias in belief-updating, learning rates were estimated separately for good and bad news trials[9,11] according to equations 11 and 12.

(11) LR_{good} = Alpha + Asymmetry

(12) LR_{bad} = Alpha - Asymmetry

The alpha parameter accounted for the tendency to learn from estimation errors independently of their valence. The asymmetry parameter indicates how much updating is biased by the valence (good/bad) of the estimation error. In more detail, the model estimated optimal alpha and asymmetry parameters per participant.

The model was implemented by using the VBA toolbox. Information was shared across all trials of a given participant, but not across participants and testing timepoints. More specifically, the toolbox uses a variational Bayes approach to approximate Bayesian inferences about parameter estimates and model comparisons. The priors for alpha and asymmetry were unbound and untransformed. The mean of the prior distribution for alpha was set to 1 and for asymmetry, zero. The model identified meaningful parameters from simulated data (see parameter recovery below).

© 2022 Bottemanne H et al. *JAMA Psychiatry*.

Model fitting details

The model was not hierarchical and the sample size of each group and timepoint did not change the values of the parameter estimates. Following[8,9] the model was fitted to each participant separately using Bayesian variational inference, yielding to a posterior distribution between parameters (for statistics: mean and variance of parameters) and a free-energy approximation for model evidence, following [10].

The individual free-energy approximations (i.e., per participant) were then fit into a random-effect Bayesian model comparison to (a) determine the probability of each participant to be best described by one of eight versions of the model (described in the Methods section) and (b) the frequency of each model version in the population to determine the model version that dominated within the population above chance. This approach formally controls for trial-to-trial variations in estimated base rates, initial estimates, and estimation error magnitude between the good news and bad news conditions, groups, and timepoints. Moreover, learning rates were calculated from optimal alpha and asymmetry components that were estimated for all trials of a given individual participant. The learning rates were calculated separately either as the sum of optimal alpha + asymmetry values for good news trials for EE > 0 or as the difference of optimal alpha – asymmetry parameters values for bad news trials for EE < 0. This means that information for alpha and asymmetry estimations were shared between all trials of a given participant, but they fit into learning rates differently as a function of the estimation error sign. Information was not shared between participants, groups, or timepoints.

© 2022 Bottemanne H et al. *JAMA Psychiatry*.

Model simulations

We checked whether the model plausibly captured asymmetric belief updating by creating surrogate data that simulated the behaviour of participants during the belief-updating task. The following parameters were used for the simulations: total trial number T = 40, alpha, asymmetry, and the number of simulations Nrep =110 per parameter setting. The alpha and asymmetry parameters were defined based on values that allowed us to best explore the parameter space qualitatively and quantitatively for the competing models. Belief-updating data was simulated for four different models: M1, with alpha = 0.6 and asymmetry = 0.1; M2, with alpha = 0.6 and asymmetry = off: M3, with alpha = off and asymmetry = 0.1; and M4, with alpha = off and asymmetry = off. M1 and M3 made similar qualitative predictions, although they differed quantitatively (eFigure 2). Notably, for M1, which took into account non-zero values for alpha and asymmetry, the belief updating was greater following positive estimation errors than following negative estimation errors than for M3, which took into account only non-zero values for asymmetry and switched alpha off. On the contrary, M2 and M4 predicted similar belief updating after positive and negative estimation errors. However, M2, which set alpha to non-zero and switched the asymmetry parameter off, predicted less overall belief updating than M4, which predicted that belief updating is proportional to the estimation error and personal relevance, without any weighting by a learning rate.

© 2022 Bottemanne H et al. *JAMA Psychiatry.*

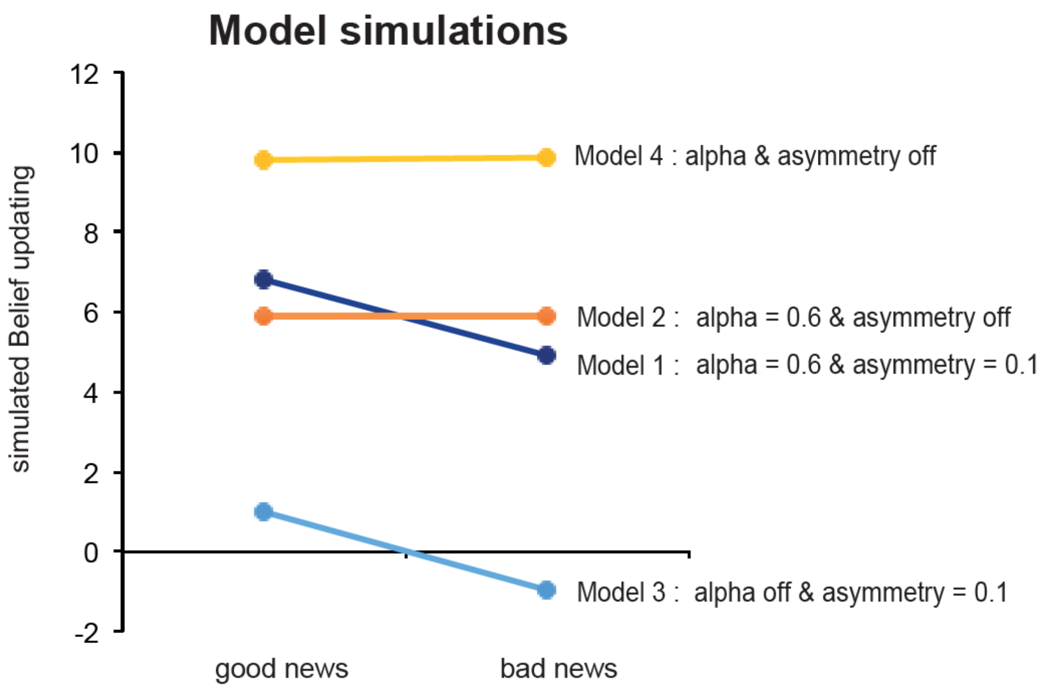

eFigure 2: Model simulations. Line graphs displaying modelled magnitudes of belief updating after good and bad news obtained from fitting four different versions of the RL model to simulated data. The four different models switched the two free parameters on and off in a 2 x 2 design.

Model comparisons

Bayesian model comparisons showed that a model that assumed asymmetrical learning from estimation errors, described best the data for all three testing time points in the TRD patients, and for the 1st assessment in the healthy controls (TRD patients T0: Ef = 0.76, pxp = 0.99; T1: Ef = 0.65, pxp = 0.95; T2: Ef = 0.63, pxp = 0.96; healthy volunteer group T0: Ef = 0.63, pxp = 0.96, eFigure 3, eTables 17 and 18). Strikingly, the belief updating data in healthy controls 2nd assessment was best described by a model that assumed asymmetrical learning and also accounted for individual differences in personal relevance of events (T1: Ef = 0.83, pxp = 0.99, eTable 18). However, this model performed not significantly better than the model that best fit the TRD patient and healthy control 1st assessment data (BIC: t(29)=-1.4, p=0.08; AIC:

© 2022 Bottemanne H et al. *JAMA Psychiatry*.

t(29)=-1.4, p =0.08, one-tailed, paired t-test). We therefore compared learning rates across groups and testing timepoints from the same model, assuming asymmetrical learning, but without the personal relevance factor.

© 2022 Bottemanne H et al. *JAMA Psychiatry*.

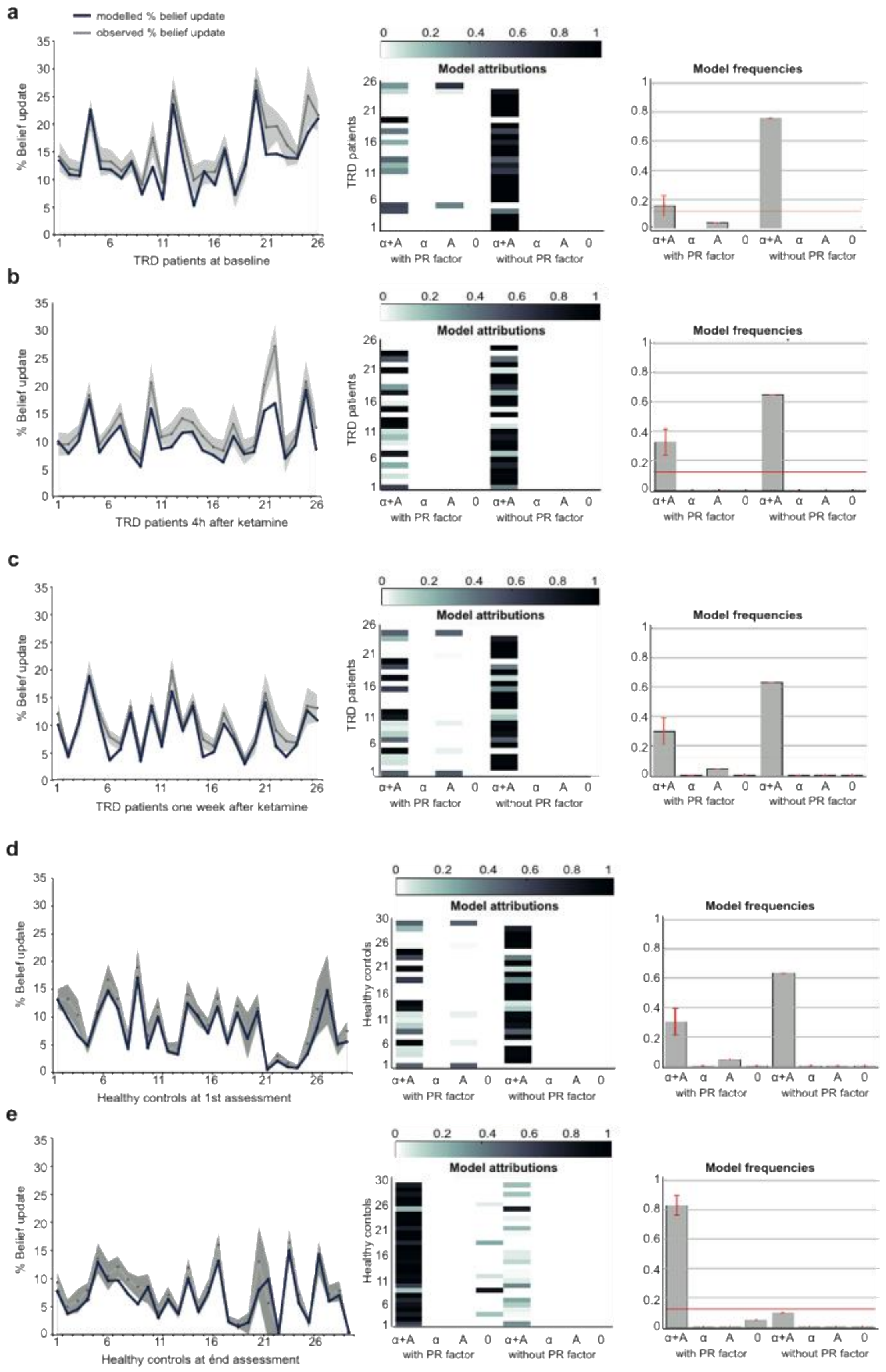

© 2022 Bottemanne H et al. *JAMA Psychiatry*.

eFigure 3: Comparisons of observed belief updates and modelled belief updates and of RL-like model versions fitted to observed belief updates. The line graphs on the left display observed (gray shades correspond to standard errors of the mean) and modelled belief updates for each participant of (a) TRD patients cohort at baseline, (b) TRD patients cohort 4h after ketamine, (c) TRD patients cohort one week after ketamine, (d) healthy control cohort at baseline (1st assessment) and (d) one week later. The histograms on the middle column panels display posterior model attributions with colored cells displaying the probability for individual participants (y axis) to be best explained by a model version (x axis). The bar graphs on the right most panels display the posterior model frequencies, which correspond to how many participants are expected to be best described by a model version with error bars corresponding to standard deviations. The red line indicates the null hypothesis that all model versions are equally likely in the cohort (chance level). Labels on the x-axis of histogram and bar graphs indicate the model versions with parameters (alpha or A) kept free. PR – personal relevance factor.

Supplementary results from computational modeling

Decomposing learning rates I: The alpha parameter

The winning model assumed asymmetrical learning from the estimation errors in terms of their valence. For this model, the learning rate involved two components: the alpha parameter assessed how much beliefs were updated proportional to the magnitude of the absolute estimation error; the asymmetry parameter assessed how much belief updating was proportional to the valence of the estimation error (positive and negative).

In accordance with the model comparisons, the average alpha parameters were significantly smaller than 1 at all testing timepoints, indicating that belief updating was

© 2022 Bottemanne H et al. *JAMA Psychiatry*.

not solely driven by the estimation error magnitude in either TRD patients (T0: alpha = 0.51 ± 0.03, t(25) = -14.3, p = 1.5e-13; T1: alpha = 0.43 ± 0.03, t(25) = -18.1, p = 7.1e-16; T2: alpha = 0.45 ± 0.04 0.07, t(25) = -14.8, p = 6.6e-14) or healthy controls (T0: alpha = 0.4 ± 0.04, t(29) = -13.7, p = 2.9e-14; T1: alpha = 0.4 ± 0.04, t(29) = -13.3, p = 6.8e-14). No differences were observed in the alpha parameter between testing timepoints for the TRD patients or controls. Such a non-difference in the alpha parameter between testing timepoints and groups indicates that the differences observed on the average learning rates before and after ketamine treatment were not driven by a deficit in learning (e.g., update beliefs) from estimation errors.

Decomposition of learning rates II: the asymmetry parameter

On the other hand, the asymmetry (A) parameter was on average significantly greater than zero at baseline (T0: A = 0.08 ± 0.02, t(29) = 3.9, p = 4.6e-04), and remained significantly greater than zero at the 2nd assessment timepoint for the healthy volunteers (T1: A = 0.07 ± 0.02, t(29) = 3.6, p = 0.001). Importantly, the asymmetry parameter was non-significant for the TRD patients before treatment (T0: A= 0.02 ± 0.02 t(25) = 0.7, p = 0.5), became significantly greater than zero after the 1st ketamine infusion (T1 : A = 0.07 ± 0.02, t(25) = 3.8, p = 7.7e-04; T0 vs T1: t(25) = -2, p = 0.05,), and remained significant one week after the 1st treatment (T2: A = 0.08 ± 0.02, t(25) = 4.1, p = 4.1e-04). This result reflects the main findings on the learning rates reported in the main manuscript.

Finally, as a sanity check, belief-updating biases correlated with the learning rate biases measured at one week of ketamine treatment for the TRD patients (eFigure 4).

© 2022 Bottemanne H et al. *JAMA Psychiatry.*

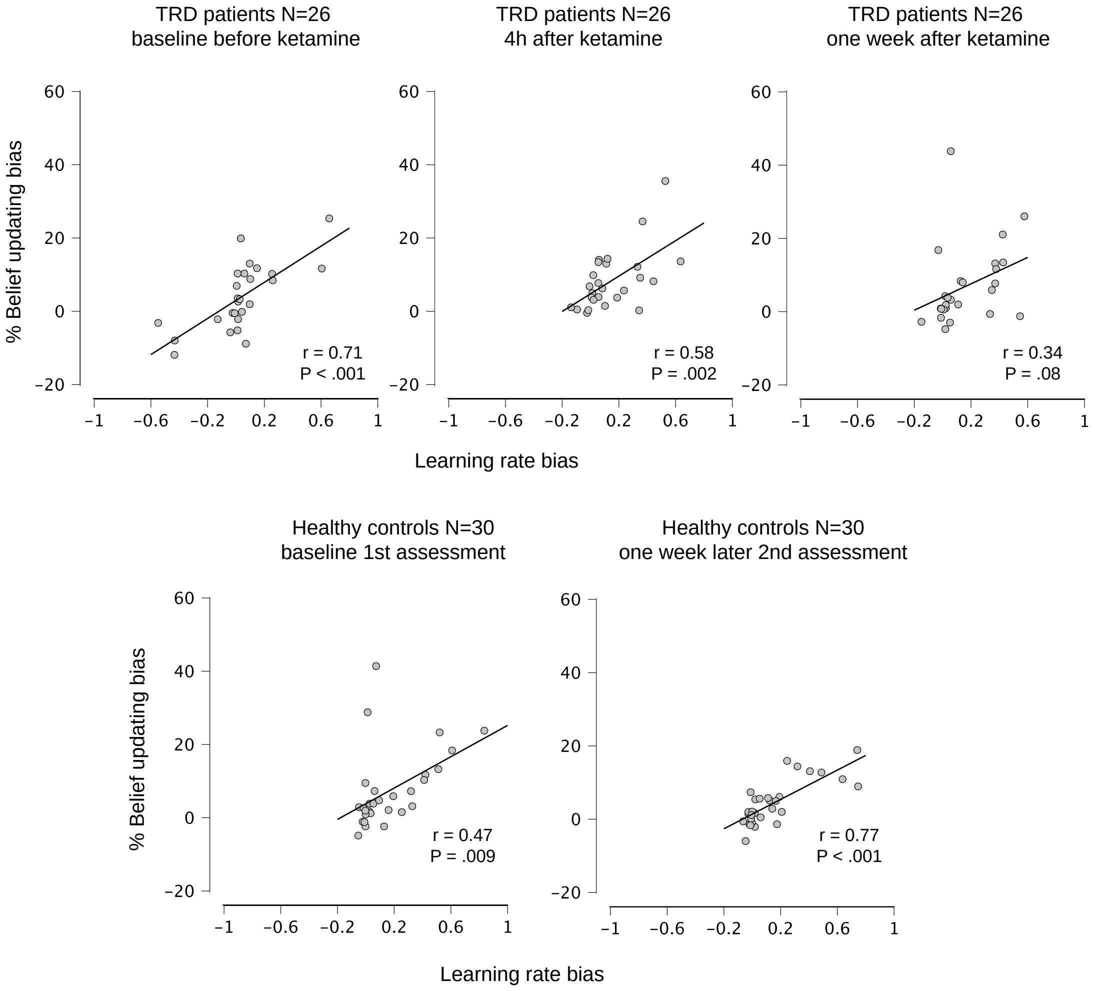

eFigure 4: Correlation between biases in learning rates and belief updating in N = 26 TRD patients. The scatterplot in the lower panel shows the covariance between the model-free observed good news/bad news update bias and the model-based bias in learning rates (LR good news – LR bad news). Each point corresponds to one patient. R indicates Pearson's correlation coefficient and p the statistically significant threshold.

Parameter recovery

We conducted a parameter recovery analysis to check whether the alpha and asymmetry parameters of the winning RL model were identifiable and described the data better than any other set of parameters.

© 2022 Bottemanne H et al. *JAMA Psychiatry*.

We first simulated belief updates using the RL model and the VBA_simulate function of the VBA toolbox. Alpha and asymmetry values to generate belief updating were randomly sampled from a Gaussian distribution, with a mean mu = 0.7 and a precision sigma = 0.2 for alpha and mu = 0.06 and sigma = 0.1 for asymmetry. Further input to the model consisted of the estimation error magnitude (EE), which was randomly sampled from a Gaussian distribution, with a mean mu = 14 and sigma = 0.95 for EE. Random numbers were generated using the VBA_random function of the VBA-toolbox. The total trial number was set to 40. The valence of the estimation errors was pseudo-randomly distributed to obtain 20 positive (good news) estimation error trials and 20 negative (bad news) estimation error trials. After simulating the data, the model was inverted to obtain the fit values for alpha and asymmetry using the VBA_StateSpaceModel function of the VBA toolbox. This process was repeated 30 times using new generating values for alpha and asymmetry. Finally, the fit and generating parameters were compared using Pearson's correlations.

For both alpha and asymmetry, the fit and generating values co-varied significantly (r_{alpha} = 0.95, p = 1.5e-16; $r_{asymmetry}$ = 0.93, p = 5.9e-04) (eFigure 5). This result indicates that the belief updates generated by the RL model with known parameters can be fit with that model to recover the parameters.

© 2022 Bottemanne H et al. *JAMA Psychiatry*.

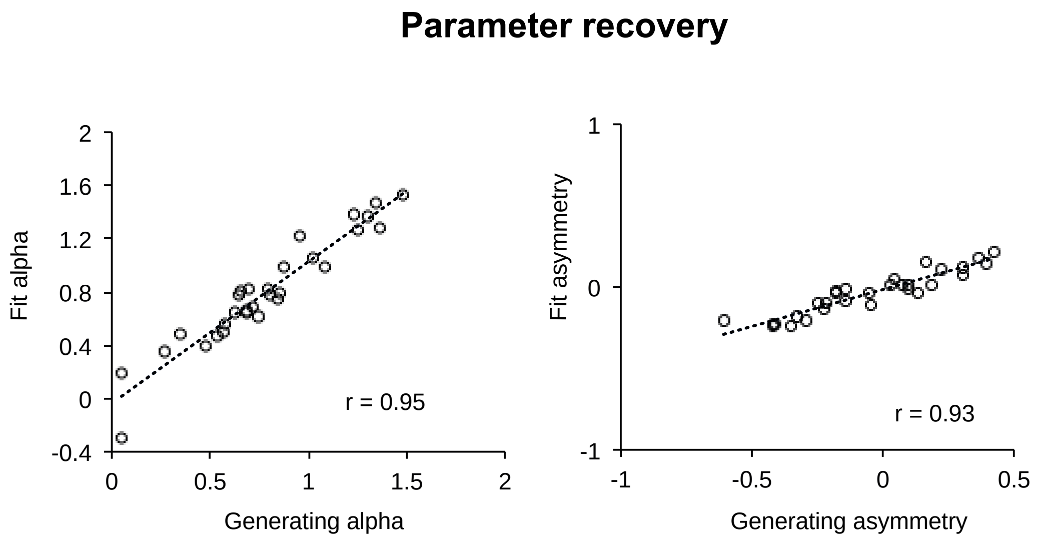

eFigure 5: Parameter recovery results. Scatterplots displaying the correlation between recovered parameters to known parameters used to generate surrogate data. Fit parameters were recovered from inverting the RL model to the surrogate data. Each dot represents one of 30 surrogate data sets, with each data set consisting of 40 trials.

© 2022 Bottemanne H et al. *JAMA Psychiatry*.

eMethods 8. Details of the Mediation Model

The model tested the following serial regression paths, controlling for the effects of the previous path at each regression:

(1) path c: $y = cx + e_y$

(2) path a: $m = ax + e_m$

(3) path b: $y = bm + c'x + e'_y$

The first regression (eq. 1), called path c, assessed the direct (or total) effect of the pharmacological challenge (*x* = testing time) on depressive symptoms (*y* = MADRS). The second regression (eq. 2), called path a, tested for an effect of the pharmacological challenge (*x* = testing time) on belief-updating biases (*m* = two scores per patient, at baseline and one week after the first ketamine infusion). This effect is equivalent to the contrast between the baseline and post-ketamine treatment belief-updating bias from a standard univariate analysis. The third regression (eq. 3), called path b, assessed the covariance between belief-updating bias (*m*) and depression symptoms (*y* = MADRS scores of each patient at baseline and one week after ketamine), controlling for the pharmacological challenge (*x* = testing time). This effect, because it controls for the effect of ketamine, tests for the endogenously driven co-variation between belief-updating bias and depression symptoms.

We conducted a single-level mediation that assessed the product of path a and path b coefficients: a * b = c – c', with c' corresponding to the effect of ketamine on depression symptoms after controlling for effects of belief-updating bias (the mediator variable).

In the mediation analysis (Figure 5), we used the normalized update bias as a mediator variable to rule out confounders due to differences in estimation error magnitude between good and bad news trials and across patients. A mediation model with a non-

© 2022 Bottemanne H et al. *JAMA Psychiatry.*

normalized update bias as a mediator (averaged across good and bad news trials for each patient) and estimation error magnitude as a covariate led to very similar results (path a: beta = 7.3, t = 2.1, p = 0.05); path b: beta = -0.3, t = -2.5, p = 0.009, and the mediation effect path a*b: beta = -2.4, t = -1.4, p=0.05).

The strong association between the MADRS scores and belief-updating biases at one week of treatment drove the mediation analysis. We split the TRD patient group into two sub-groups: a group of six TRD patients who showed remission (n = 1) or treatment responsiveness (n = 5) after one week of treatment and a second group of 20 TRD patients who did not show treatment responsiveness or remission after one week of treatment. We then tested for early belief-updating biases 4 h after the first ketamine infusion within the group of 20 TRD patients who did not display any clinical responsiveness or remission after one week of treatment. The results show that although this group remained clinically unresponsive after one week of treatment, they already showed a significant increase in optimistically biased belief updating (bias 4 h after ketamine – baseline = 4 ± 2% increase, t(19) = 2, p = 0.03). These results provide evidence that patients showed a change in optimistically biased belief updating even before clinical responsiveness was observed on the MADRS.

© 2022 Bottemanne H et al. *JAMA Psychiatry*.

eMethods 9. Task Instructions in French

The patients were told the following before performing the belief-updating task:

« Il s'agit d'un test qui mesure vos croyances concernant des évènements de vie future.

40 événements de vie seront présentés successivement. Vous devrez pour chacun d'entre eux donner :

1. La probabilité qu'il se produise dans votre vie future,
2. La confiance que vous avez dans votre estimation,
3. La probabilité qu'il se produise pour quelqu'un d'autre.

On vous montrera alors le taux officiel que cet évènement se produise dans la société. On vous demandera de noter votre confiance dans le taux officiel si vous pensez qu'il est correct ou qu'on s'est trompé dans les sondages.

Puis vous devrez pour chacun d'entre eux donner à nouveau :

1. La probabilité qu'il se produise dans votre vie future,
2. La confiance que vous avez dans votre estimation.

Merci de ne pas donner exactement les mêmes probabilités à chaque estimation. »

eTable 1. Demographics

	Healthy controls (n=30)	TRD patients (n=26)
Sex, male : female	13 : 17	16 : 10
Years of age	52.2 ± 1.7 (22 – 59)	52.5 ± 1.9 (34 – 67)
Years of higher education	3.9 ± 0.3 (0 – 5)	3.9 ± 0.4 (0 – 5)
Total MADRS score	0.3 ± 0.2	37.1 ± 1.4

mean ± sem (range), TRD (treatment resistant depression) patients. Both groups were non-different for male participant distribution ($X^2 = 3.1$, $p = 0.08$), and female participant distribution ($X^2 = 3.3$, $p = 0.06$). The level of education was calculated as the years of study after high school.

© 2022 Bottemanne H et al. *JAMA Psychiatry*.

eTable 2. Clinical Characteristics of TRD Patients (n = 26)

Years of current episode (mean ± sem)	5.7 ± 0.8
Age at onset of illness (mean ± sem)	31.0 ± 2.3
Unipolar : bipolar depression	20 : 6
Total MADRS score (mean ± sem)	37.1 ± 1.4
Total MSM score (mean ± sem)	9.4 ± 0.4
**Disease severity, MADRS category**	
Severe depression n (%)	20 (77%)
Moderate depression n (%)	6 (23%)
**Resistance severity, MSM category**	
Severe resistance n (%)	12 (46%)
Moderate resistance n (%)	14 (54%)
**Treatments (% of 26)**	
SSRIs	69%
SNRIs	19%
Tricyclic	8%
Antipsychotic	12%
Mood stabilizer	46%
Benzodiazepine	54%
**Anticipated treatment outcome (mean % ratings ± sem)**	
Expected drug efficiency	53.1 ± 5.6
Expected response	33.6 ± 4.9
Expectation of remission	33.8 ± 5.1

MSM: Maudsley Staging Method, MADRS: Montgomery-Asberg Depression Rating Scale, SSRI: selective serotonin reuptake inhibitors, SNRIs: serotonin and noradrenalin reuptake inhibitors, sem: standard error of the mean. The years of current episode was defined as the duration of the current depressive episode. The age at onset of illness was defined as the age (years) at diagnosis with MDD. Disease severity was defined according to the MADRS score: moderate (20 to 34), severe (35 to 60). Resistance severity was defined according to the MSM score: moderate (7 to 10), severe (11 to 15).

© 2022 Bottemanne H et al. *JAMA Psychiatry*.

eTable 3. Improvement in Depression Measured by the MADRS Before and After Ketamine Treatment for 26 TRD Patients

	Median (IQR)			Statistics	
	**T0**	**T1**	**T2**	**T0 > T1**	**T0 > T2**
Total MADRS	37 (9)	33 (10)	27 (16)	P = 0.001, z = 3.3	P = 4.1e-05, z = 4.1
Sadness	4.5 (1)	4 (2)	3 (2)	P = 0.02, z = 2.2	P = 2.7e-04, z = 3.6
Neurovegetative	4 (1)	3 (1)	2 (2)	P = 7e-04, z = 3.4	P = 1.1e-04, z = 3.8
Detachment	4 (2)	4 (1)	3.5 (1)	P = 0.08, z = 1.7	P = 0.003, z = 2.9
Negative thoughts	3.5 (1)	3 (2)	3 (1)	P = 0.004, z = 2.8	P = 8.2e-04, z = 3.3

MADRS: Montgomery-Asberg Depression Rating Scale, IQR: interquartile range, T0: baseline measurement 24 h before the first ketamine infusion, T1: 4 h after first ketamine infusion, T2: 4 h after the third ketamine infusion one week after T1. Reported statistics are for paired Wilcoxon rank tests.

© 2022 Bottemanne H et al. *JAMA Psychiatry*.

eTable 4. Average Number of Trials Excluded From Analyses of the Belief-Updating Task

	**Healthy controls**		**TRD patients**		
	T0	T1	T0	T1	T2
**EE = 0**	1 ± 0.2	0.9 ± 0.2	0.5 ± 0.2	1.5 ± 0.3	1.65 ± 0.24
**Paradoxical updating**	4.2 ± 0.5	5.7 ± 0.8	4.7 ± 0.6	5.7 ± 0.6	5.2 ± 0.6

The number of excluded trials did not differ significantly between groups or testing timepoints using paired and two-sampled, two-tailed t-tests. Paradoxical updating was defined by a higher second estimate (E2) relative to the first estimate (E1) (E1 < E2), despite good news. Specifically, in these trials, the base rate (BR) indicated that participants had overestimated their likelihood of experiencing an adverse life event (E1 > BR). Paradoxical updating also occurred when E2 of participants further decreased (E1 > E2), despite bad news (E1 < BR). The base rate (BR) indicated they had underestimated their likelihood of experiencing an adverse life event.

© 2022 Bottemanne H et al. *JAMA Psychiatry*.

eTable 5. Belief-Updating Task Variables

		TRD patients N = 26			Controls N = 30	
		**T0**	**T1**	**T2**	**T0**	**T1**
Prior estimate (E1)	EE > 0	62 ± 3	60 ± 3	52 ± 2	59 ± 2	53 ± 2
	EE < 0	19 ± 1	20 ± 1	21 ± 1	18 ± 1	20 ± 1
Estimation error	EE > 0	28 ± 2	26 ± 2	20 ± 2	25 ± 1	21 ± 1
	EE < 0	26 ± 1	25 ± 1	23 ± 1	25 ± 1	245± 1
Personal relevance	EE > 0	0.7 ± 0.03	0.7 ± 0.02	0.8 ± 0.02	0.7 ± 0.03	0.7 ± 0.02
	EE < 0	0.6 ± 0.03	0.6 ± 0.03	0.7 ± 0.04	0.8 ± 0.02	0.7 ± 0.03
Confidence E1	EE > 0	72 ± 3	73 ± 3	69 ± 4	70 ± 4	66 ± 6
	EE < 0	67 ± 3	70 ± 3	68 ± 4	66± 4	59 ± 7
Confidence E2	EE > 0	74 ± 3	75 ± 2	69 ± 4	68 ± 7	68 ± 7
	EE < 0	73 ± 3	73 ± 3	70 ± 4	67 ± 6	64 ± 6
Confidence TBR	EE > 0	72 ± 3	74 ± 3	78 ± 3	73 ± 3	73 ± 3
	EE < 0	73 ± 3	75 ± 3	78 ± 3	73 ± 2	72 ± 3

© 2022 Bottemanne H et al. *JAMA Psychiatry*.

eTable 6. Linear Mixed-Effects Model Results Fitting the Average Absolute Belief Updates (UPD) Before (T0) and 4 h After the First Ketamine Infusion (T1)

|UPD|~ 1 + group + time + valence + |EE |+ age + education + group:time:valence + (1+valence | SUB) + (1+|EE| | SUB)							
**Model fit statistics:**	**AIC**	**BIC**	**LL**	**Deviance**	**Ordinary R^2**	**Adjusted R^2**	
	**1523**	**1577**	**-745**	**1491**	**0.74**	**0.73**	
**Fixed effects**						**95%CI**	
**Name**	**Beta estimate**	**SE**	**tvalue**	**DF**	**p**	**Lower**	**Upper**
**Intercept**	15.9	5.3	3.03	216	0.003	5.6	26.4
**group**	-0.43	0.75	-0.56	216	0.57	-1.9	1.06
**time**	-0.78	0.35	-2.26	216	0.02	-1.5	-0.1
**valence**	3.02	0.45	6.74	216	1.4^{e}-10	2.1	3.9
**|EE|**	0.08	0.08	1.05	216	0.29	-0.07	0.23
**Age**	-0.03	0.08	-0.32	216	0.74	-0.2	0.13
**Education**	-0.83	0.44	-1.89	216	0.06	-1.7	0.03
**Group*Time*Valence**	-0.91	0.34	-2.67	216	0.008	-1.6	-0.2
**Group : Subject (56 levels)**			Beta	Lower	Upper		
**Intercept**	Intercept	std	1.6^{e}-06	NaN	NaN		
**Group : Subject (56 levels)**			Beta	Lower	Upper		
**Intercept**	Intercept	std	4.9	3.2	7.6		
**valence**	Intercept	corr	1	NaN	NaN		
**valence**	valence	std	2.1	1 .3	3.4		
**Group : Subject (56 levels)**			Beta	Lower	Upper		
**Intercept**	Intercept	std	2.8	0.5	16.8		
**|EE|**	Intercept	corr	-1	NaN	NaN		
**|EE|**	valence	std	0.25	0.13	30.52		

© 2022 Bottemanne H et al. *JAMA Psychiatry*.

eTable 7. Linear Mixed-Effects Model Results Fitting the Average Absolute Belief Updates (UPD) Before (T0) and 1 Week After the First Ketamine Infusion (T2)

|UPD|~ 1 + group + time + valence + |EE |+ age + education + group:time:valence + (1+valence | SUB) + (1+|EE| | SUB)							
**Model fit statistics:**	**AIC**	**BIC**	**LL**	**Deviance**	**Ordinary R^2**	**Adjusted R^2**	
	**1534**	**1589**	**-751**	**1502**	**0.75**	**0.74**	
**Fixed effects**						**95%CI**	
**Name**	**Beta estimate**	**SE**	**tvalue**	**DF**	**p**	**Lower**	**Upper**
**Intercept**	10.3	5.2	1.97	216	0.05	0.002	20.6
**group**	-0.4	0.75	-0.52	216	0.61	-1.8	1.09
**time**	-0.65	0.37	-1.75	216	0.08	-1.4	0.08
**valence**	2.9	0.51	5.89	216	1.4^{e}-08	1.9	3.9
**|EE|**	0.21	0.08	2.58	216	0.01	0.05	0.37
**Age**	-0.02	0.08	0.24	216	0.81	-0.14	0.18
**Education**	-0.81	0.43	-1.88	216	0.06	-1.6	0.04
**Group*Time*Valence**	-0.73	0.35	-2.11	216	0.03	-1.4	-0.04
**Group : Subject (56 levels)**			Beta	Lower	Upper		
**Intercept**	Intercept	std	1.36^{e}-06	NaN	NaN		
**Group : Subject (56 levels)**			Beta	Lower	Upper		
**Intercept**	Intercept	std	4.5	3.2	6.3		
**valence**	Intercept	corr	1	NaN	NaN		
**valence**	valence	std	2.7	1.9	3.6		
**Group : Subject (56 levels)**			Beta	Lower	Upper		
**Intercept**	Intercept	std	2.1	0.2	19.8		
**|EE|**	Intercept	corr	-1	NaN	NaN		
**|EE|**	valence	std	0.26	0.14	0.48		

© 2022 Bottemanne H et al. *JAMA Psychiatry*.

eTable 8. Linear Mixed-Effects Model Results Fitting the Average Initial Belief Estimates (E1) at Baseline and 4 h After Ketamine Treatment

E1 |~ 1 + group + time + valence + age + education + group:time:valence + (1+valence | SUB)							
**Model fit statistics:**	**AIC**	**BIC**	**LL**	**Deviance**	**Ordinary R^2**	**Adjusted R^2**	
	**1601**	**1641**	**-788**	**1576**	**0.92**	**0.92**	
**Fixed effects**						**95%CI**	
**Name**	**Beta estimate**	**SE**	**tvalue**	**DF**	**p**	**Lower**	**Upper**
**Intercept**	40.1	3.27	12.2	216	1.45^{e}-26	33.7	46.6
**group**	-1.3	0.51	-2.64	216	0.008	-2.3	0.35
**time**	-0.75	0.44	-1.7	216	0.09	-1.6	0.12
**valence**	18.7	0.94	19.8	216	1.03^{e}-50	16.8	20.6
**Age**	-0.02	0.06	-0.37	216	0.71	-0.13	0.08
**Education**	-0.02	0.28	-0.07	216	0.94	-0.58	0.54
**Group*Time*Valence**	-0.75	0.45	-1.68	216	0.09	-1.63	0.13
**Group : Subject (56 levels)**			Beta	Lower	Upper		
**Intercept**	Intercept	std	3.8	2.7	5.2		
**valence**	Intercept	corr	1	NaN	NaN		
**valence**	valence	std	6.2	4.8	7.8		

© 2022 Bottemanne H et al. *JAMA Psychiatry*.

eTable 9. Linear Mixed-Effects Model Results Fitting the Personal Relevance of Events At Baseline and 4 h After Ketamine Treatment

PR ~ 1 + group + time + valence +age + education + |EE| + group:time:valence + (1+valence | SUB) + (1+|EE| | SUB)							
**Model fit statistics:**	**AIC**	**BIC**	**LL**	**Deviance**	**Ordinary R^2**	**Adjusted R^2**	
	**-189**	**-135**	**111**	**-222**	**0.25**	**0.22**	
**Fixed effects**						**95%CI**	
**Name**	**Beta estimate**	**SE**	**tvalue**	**DF**	**p**	**Lower**	**Upper**
**Intercept**	0.8	0.08	9.50	216	4.03^{e}-18	0.64	0.98
**group**	0.03	0.01	-2.31	216	0.02	0.004	0.05
**time**	0.01	0.009	-1.17	216	0.23	-0.007	0.03
**valence**	0.04	0.009	3.75	216	0.0002	0.01	0.05
**|EE|**	-0.004	0.001	-2.72	216	0.006	-0.006	-0.001
**Age**	-0.0002	0.001	-0.15	216	0.88	-0.002	0.002
**Education**	-0.0007	0.007	-0.11	216	0.91	-0.01	0.01
**Group*Time*Valence**	-0.006	0.009	-0.71	216	0.47	-0.02	0.01
**Group : Subject (56 levels)**			Beta	Lower	Upper		
**Intercept**	Intercept	std	0.04	0.01	0.16		
**valence**	Intercept	corr	-1	NaN	NaN		
**valence**	valence	std	0.01	0.004	0.09		
**Group : Subject (56 levels)**							
**Intercept**	Intercept	std	0.01	3.9	2704.9		
**|EE|**	Intercept	corr	-1	NaN	NaN		
**|EE|**	|EE|	std	0.002	0.0003	0.014		

© 2022 Bottemanne H et al. *JAMA Psychiatry*.

eTable 10. Linear Mixed-Effects Model Results Fitting Absolute Estimation Error Magnitude at Baseline and 4 h After Ketamine Treatment

|EE| ~ 1 + group + time + valence +age + education + group:time:valence + (1+valence | SUB)							
**Model fit statistics:**	**AIC**	**BIC**	**LL**	**Deviance**	**Ordinary R^2**	**Adjusted R^2**	
	**1486**	**1527**	**-731**	**1462**	**0.58**	**0.57**	
**Fixed effects**						**95%CI**	
**Name**	**Beta estimate**	**SE**	**tvalue**	**DF**	**p**	**Lower**	**Upper**
**Intercept**	27	3.53	7.6	217	7.22^{e}-13	20.03	33.9
**group**	-0.5	0.55	-0.89	217	0.37	-1.58	0.59
**time**	-1.1	0.32	-3.48	217	0.0006	-1.77	-0.49
**valence**	-0.36	0.54	-0.65	217	0.51	-1.44	0.72
**Age**	-0.03	0.06	-0.51	217	0.61	-0.15	0.08
**Education**	-0.1	0.30	-0.34	217	0.73	-0.71	0.50
**Group*Time*Valence**	-0.43	0.32	-1.34	217	0.18	-1.1	0.20
**Group : Subject (56 levels)**			Beta	Lower	Upper		
**Intercept**	Intercept	std	3.3	NaN	NaN		
**valence**	Intercept	corr	0.88	NaN	NaN		
**valence**	valence	std	3.31	2.5	4.4		

© 2022 Bottemanne H et al. *JAMA Psychiatry*.

eTable 11. Linear Mixed-Effects Model Results Fitting Confidence Ratings in the Base Rates of Events at Baseline and 4 h After Ketamine Treatment

Confidence BR ~ 1 + group + time + valence +age + education + |EE| + group:time:valence + (1+valence | SUB) + (1+|EE| | SUB)							
**Model fit statistics:**	**AIC**	**BIC**	**LL**	**Deviance**	**Ordinary R^2**	**Adjusted R^2**	
	**1892**	**1946**	**-930**	**1860**	**0.46**	**0.44**	
**Fixed effects**						**95%CI**	
**Name**	**Beta estimate**	**SE**	**tvalue**	**DF**	**p**	**Lower**	**Upper**
**Intercept**	92.4	11.8	7.8	216	2.5^{e}-13	69.1	115.7
**group**	-0.10	1.7	-0.06	216	0.95	-3.6	3.35
**time**	-0.73	0.87	-0.83	216	0.40	-2.5	0.99
**valence**	-0.08	0.86	-0.09	216	0.92	-1.8	1.62
**|EE|**	-0.26	0.15	-1.79	216	0.07	-0.56	0.03
**Age**	-0.35	0.19	-1.8	216	0.07	-0.73	0.03
**Education**	1.25	0.98	1.3	216	0.20	-0.67	3.2
**Group*Time*Valence**	-0.12	0.86	-0.14	216	0.88	-1.82	1.6
**Group : Subject (56 levels)**			Beta	Lower	Upper		
**Intercept**	Intercept	std	9.56	0.1	891		
**valence**	Intercept	corr	1	NaN	NaN		
**valence**	valence	std	0.15	0.002	14.5		
**Group : Subject (56 levels)**							
**Intercept**	Intercept	std	4.5	NaN	NaN		
**|EE|**	Intercept	corr	1	NaN	NaN		
**|EE|**	|EE|	std	0.06	NaN	NaN		

© 2022 Bottemanne H et al. *JAMA Psychiatry*.

eTable 12. Linear Mixed-Effects Model Results Fitting the Update Bias at Baseline and 4 h After Ketamine Treatment

UDB ~ 1 + group + time +age + education + |EE| + group:time + (1+|EE| | SUB)							
**Model fit statistics:**	**AIC**	**BIC**	**LL**	**Deviance**	**Ordinary R^2**	**Adjusted R^2**	
	**797**	**829**	**-386**	**773**	**0.32**	**0.28**	
**Fixed effects**						**95%CI**	
**Name**	**Beta estimate**	**SE**	**tvalue**	**DF**	**p**	**Lower**	**Upper**
**Intercept**	-21.3	6.5	-3.3	105	0.001	-34.3	-8.4
**group**	0.83	0.76	1.08	105	0.29	-0.68	2.34
**time**	0.93	0.68	1.35	105	0.17	-0.42	2.29
**|EE|**	0.61	0.17	3.6	105	0.0005	0.27	0.94
**Age**	0.23	0.08	2.6	105	0.009	0.05	0.39
**Education**	0.15	0.42	0.4	105	0.71	-0.68	0.98
**Group*Time**	-1.65	0.67	-2.5	105	0.01	-2.9	-0.32
**Group : Subject (56 levels)**							
**Intercept**	Intercept	std	9.33	8.35	10.4		
**|EE|**	Intercept	corr	-1	NaN	NaN		
**|EE|**	|EE|	std	0.48	0.43	0.54		

© 2022 Bottemanne H et al. *JAMA Psychiatry*.

eTable 13. Linear Mixed-Effects Model Results Fitting Belief Updates, Including Paradoxical Trials, at Baseline and 4 h After Ketamine Treatment

UPDpara ~ 1 + group + time + valence +age + education + |EEpara| + group:time:valence + (1+valence | SUB) + (1+|EEpara| | SUB)							
**Model fit statistics:**	**AIC**	**BIC**	**LL**	**Deviance**	**Ordinary R^2**	**Adjusted R^2**	
	**1553**	**1607**	**-760**	**1521**	**0.56**	**0.54**	
**Fixed effects**						**95%CI**	
**Name**	**Beta estimate**	**SE**	**tvalue**	**DF**	**p**	**Lower**	**Upper**
**Intercept**	7.2	3.7	3.7	216	0.06	-0.2	14.6
**group**	-0.87	0.5	0.5	216	0.07	-1.8	0.06
**time**	-0.88	0.4	0.4	216	0.02	-1.6	-0.12
**valence**	2.3	0.73	0.7	216	0.002	0.87	3.7
**|EE|**	0.17	0.08	0.08	216	0.05	0.001	0.34
**Age**	0.007	0.05	0.05	216	0.89	-0.09	0.11
**Education**	-0.57	0.27	0.3	216	0.03	-1.1	-0.04
**Group*Time*Valence**	-0.79	0.38	0.4	216	0.04	-1.5	-0.03
**Group : Subject (56 levels)**			Beta	Lower	Upper		
**Intercept**	Intercept	std	0.65	0.51	0.83		
**valence**	Intercept	corr	-1	NaN	NaN		
**valence**	valence	std	4.61	3.54	0.83		
**Group : Subject (56 levels)**							
**Intercept**	Intercept	std	3.85	2.81	5.28		
**|EE|**	Intercept	corr	-1	NaN	NaN		
**|EE|**	|EE|	std	0.25	0.18	0.33		

© 2022 Bottemanne H et al. *JAMA Psychiatry*.

eTable 14. Linear Mixed-Effects Model Results Fitting Learning Rates at Baseline

LR |~ 1 + group + valence + age + education + group:valence + (1+valence | SUB)							
**Model fit statistics:**	**AIC**	**BIC**	**LL**	**Deviance**	**Ordinary R^2**	**Adjusted R^2**	
	**-3.3**	**26**	**12.7**	**-25**	**0.92**	**0.91**	
**Fixed effects**						**95%CI**	
**Name**	**Beta estimate**	**SE**	**tvalue**	**DF**	**p**	**Lower**	**Upper**
**Intercept**	0.52	0.16	3.13	106	0.002	0.2	0.85
**group**	-0.05	0.02	-2.1	106	0.04	-0.1	-0.002
**valence**	0.05	0.01	3.1	106	0.002	0.02	0.08
**Age**	0.001	0.003	0.5	106	0.64	-0.004	0.007
**Education**	-0.04	0.01	-2.5	106	0.01	-0.06	-0.007
**Group*Valence**	0.03	0.01	2.03	106	0.04	0.0008	0.06
**Group : Subject (56 levels)**			Beta	Lower	Upper		
**Intercept**	Intercept	std	0.12	NaN	NaN		
**valence**	Intercept	corr	0.24	NaN	NaN		
**valence**	valence	std	0.08	NaN	NaN		

© 2022 Bottemanne H et al. *JAMA Psychiatry*.

eTable 15. Linear Mixed-Effects Model Results Fitting Learning Rates 4 h After the First Ketamine Infusion

LR |~ 1 + group + valence + age + education + group:valence + (1+valence | SUB)							
**Model fit statistics:**	**AIC**	**BIC**	**LL**	**Deviance**	**Ordinary R^2**	**Adjusted R^2**	
	**-26.4**	**3.5**	**24.2**	**-48.4**	**0.92**	**0.92**	
**Fixed effects**						**95%CI**	
**Name**	**Beta estimate**	**SE**	**tvalue**	**DF**	**p**	**Lower**	**Upper**
**Intercept**	0.59	0.15	3.8	106	0.002	0.3	0.9
**group**	-0.01	0.03	-0.6	106	0.52	-0.07	0.04
**valence**	0.07	0.01	5.3	106	5.9e-07	0.04	0.1
**Age**	-0.002	0.003	-0.8	106	0.4	-0.007	0.003
**Education**	-0.02	0.01	-1.3	106	0.21	-0.04	0.009
**Group*Valence**	0.002	0.01	0.12	106	0.9	-0.03	0.03
**Group : Subject (56 levels)**			Beta	Lower	Upper		
**Intercept**	Intercept	std	0.19	NaN	NaN		
**valence**	Intercept	corr	0.67	NaN	NaN		
**valence**	valence	std	0.08	NaN	NaN		

© 2022 Bottemanne H et al. *JAMA Psychiatry*.

eTable 16. Linear Mixed-Effects Model Results Fitting Learning Rates 1 Week After the First Ketamine Infusion

LR |~ 1 + group + valence + age + education + group:valence + (1+valence | SUB)							
**Model fit statistics:**	**AIC**	**BIC**	**LL**	**Deviance**	**Ordinary R^2**	**Adjusted R^2**	
	**-19.5**	**10.4**	**20.7**	**-41.5**	**0.93**	**0.93**	
**Fixed effects**						**95%CI**	
**Name**	**Beta estimate**	**SE**	**tvalue**	**DF**	**p**	**Lower**	**Upper**
**Intercept**	0.70	0.16	4.4	106	2.9e-05	0.4	1
**group**	-0.03	0.03	-0.9	106	0.34	-0.08	0.03
**valence**	0.08	0.01	5.5	106	2.6e-07	0.05	0.1
**Age**	-0.003	0.003	-1.4	106	0.15	-0.009	0.001
**Education**	-0.02	0.01	-1.3	106	0.19	-0.05	0.009
**Group*Valence**	-0.002	0.01	0.14	106	0.8	-0.03	0.03
**Group : Subject (56 levels)**			Beta	Lower	Upper		
**Intercept**	Intercept	std	0.19	NaN	NaN		
**valence**	Intercept	corr	0.64	NaN	NaN		
**valence**	valence	std	0.08	NaN	NaN		

© 2022 Bottemanne H et al. *JAMA Psychiatry*.

eTable 17. Model Comparisons TRD Patients

TRD patients	Baseline (T0				4h after ketamine (T1)				1 week after ketamine (T2)			
	**Ef**	**pxp**	**BIC**	**AIC**	**Ef**	**pxp**	**BIC**	**AIC**	**Ef**	**pxp**	**BIC**	**AIC**
Model 1	0.16	0.0003	-147	-145	0.33	0.04	-137	-136	0.31	0.03	-134	-133
Model 2	0.004	0	-146	-146	0.005	0	-137	-137	0.005	0	-134	-133
Model 3	0.05	0	-149	-148	0.005	0	-144	-143	0.04	0	-137	-136
Model 4	0.005	0	-149	-149	0.005	0	-144	-144	0.005	0	-141	-141
**Model 5**	**0.76**	**0.99**	**-142**	**-141**	**0.64**	**0.96**	**-135**	**-134**	**0.63**	**0.96**	**-132**	**-130**
Model 6	0.005	0	-143	-143	0.005	0	-136	-135	0.005	0	-133	-132
Model 7	0.005	0	-155	-154	0.005	0	-152	-151	0.005	0	-146	-145
Model 8	0.005	0	-157	-157	0.005	0	-154	-154	0.005	0	-152	-152

© 2022 Bottemanne H et al. *JAMA Psychiatry*.

eTable 18. Model Comparisons Healthy Controls

**Healthy controls**	**1st assessment baseline (T0**				**2nd assessment 1 week later (T1)**			
	**Ef**	**pxp**	**BIC**	**AIC**	**Ef**	**pxp**	**BIC**	**AIC**
Model 1	0.30	0,0329	-137	**-136**	**0.83**	**0.99**	**-132**	**-131**
Model 2	0.005	0	-138	-138	0.004	0	-133	-133
Model 3	0.05	0	-152	-152	0.004	0	-147	-146
Model 4	0.005	0	-154	-154	0.052	0	-151	-151
**Model 5**	**0.63**	**0.96**	**-134**	**-135**	0.10	0	-135	-134
Model 6	0.005	0	-134	-138	0.004	0	-136	-135
Model 7	0.005	0	-150	-161	0.004	0	-159	-158
Model 8	0.005	0	-155	-166	0.004	0	-166	-166

Ef – estimated model frequency that reflects how frequent each model is in each cohort ; pxp – exceedance probability, which reflects the probability of that model to be predominant in the cohort above chance. BIC – Bayesian information criterion, AIC – Aikaike information criterium

© 2022 Bottemanne H et al. *JAMA Psychiatry*.

eReferences.

1. 1. Han Y, Chen J, Zou D, et al. Efficacy of ketamine in the rapid treatment of major depressive disorder: a meta-analysis of randomized, double-blind, placebo-controlled studies. *Neuropsychiatr Dis Treat.* 2016;12:2859-2867. doi:10.2147/NDT.S117146
2. 2. Romeo B, Choucha W, Fossati P, Rotge JY. Meta-analysis of short- and mid-term efficacy of ketamine in unipolar and bipolar depression. *Psychiatry Res.* 2015;230(2):682-688. doi:10.1016/j.psychres.2015.10.032
3. Quilty LC, Robinson JJ, Rolland J, Fruyt FD, Rouillon F, Bagby RM. The structure of the Montgomery–Åsberg depression rating scale over the course of treatment for depression. *Int J Methods Psychiatr Res*. 2013; 22(3):175–84.
4. Kirsch I, Sapirstein G. Listening to Prozac but hearing placebo: A meta-analysis of antidepressant medication. *Prev Treat.* 1998;1(2): No Pagination Specified-No Pagination Specified.
5. Kirsch I. Response Expectancy and the Response to Antidepressant Medication. *EBioMedicine.* 2017; 25:13.
6. Korn CW, Sharot T, Walter H, Heekeren HR, Dolan RJ. Depression is related to an absence of optimistically biased belief updating about future life events. *Psychol Med.* 2014; 44(3):579-592.
7. Garrett N, Sharot T, Faulkner P, Korn CW, Roiser JP, Dolan RJ. Losing the rose tinted glasses: neural substrates of unbiased belief updating in depression. *Front Hum Neurosci.* 2014;8. doi:10.3389/fnhum.2014.00639
8. Kuzmanovic B, Rigoux L. Valence-dependent belief updating: computational validation. Front Psychol 2017; 8:1087.
9. Kuzmanovic B, Rigoux L, Tittgemeyer M. Influence of vmPFC on dmPFC Predicts Valence-Guided Belief Formation. *J Neurosci Off J Soc Neurosci*. 2018;38(37):7996-8010. doi:10.1523/JNEUROSCI.0266-18.2018
10. Rigoux L, Stephan KE, Friston KJ, Daunizeau J. Bayesian model selection for group studies - revisited. *NeuroImage.* 2014;84:971-985. doi:10.1016/j.neuroimage.2013.08.065
11. Lefebvre G, Lebreton M, Meyniel F, Bourgeois-Gironde S, Palminteri S. Behavioural and neural characterization of optimistic reinforcement learning. *Nat Hum Behav*. 2017;1(4):1-9. doi:10.1038/s41562-017-0067

© 2022 Bottemanne H et al. *JAMA Psychiatry*.
